# Altered Gut Microbiota as a Potential Risk Factor for Coronary Artery Disease in Diabetes: A Two-Sample Bi-Directional Mendelian Randomization Study

**DOI:** 10.7150/ijms.92131

**Published:** 2024-01-01

**Authors:** Zhaopei Zeng, Junxiong Qiu, Yu Chen, Diefei Liang, Feng Wei, Yuan Fu, Jiarui Zhang, Xiexiao Wei, Xinyi Zhang, Jun Tao, Liling Lin, Junmeng Zheng

**Affiliations:** 1 Department of Cardiovascular Surgery, Sun Yat-sen Memorial Hospital, Sun Yat-sen University, Guangzhou, China.; 2 Guangdong Provincial Key Laboratory of Malignant Tumor Epigenetics and Gene Regulation, Sun Yat-sen Memorial Hospital, Sun Yat-sen University, Guangzhou, China.; 3 Department of Surgical Oncology, The First Affiliated Hospital, School of Medicine, Zhejiang University, Hangzhou, China.; 4 Department of Endocrinology, Sun Yat-sen Memorial Hospital, Sun Yat-sen University, Guangzhou, China.; 5 Department of Cardiothoracic Surgery, Shenshan Medical Center, Sun Yat-sen Memorial Hospital, Sun Yat-sen University, Shanwei, China.; 6 Department of Cardiology, Chinese PLA General Hospital, Beijing, China.; 7 Department of Anesthesiology, Sun Yat-sen Memorial Hospital, Sun Yat-sen University, Guangzhou, China.

**Keywords:** coronary artery disease, type 2 diabetes, causality, gut microbiota, metabolites, Mendelian randomization

## Abstract

The current body of research points to a notable correlation between an imbalance in gut microbiota and the development of type 2 diabetes mellitus (T2D) as well as its consequential ailment, coronary artery disease (CAD). The complexities underlying the association, especially in the context of diabetic coronary artery disease (DCAD), are not yet fully understood, and the causal links require further clarification. In this study, a bidirectional Mendelian randomization (MR) methodology was utilized to explore the causal relationships between gut microbiota, T2D, and CAD. By analyzing data from the DIAGRAM, GERA, UKB, FHS, and mibioGen cohorts and examining GWAS databases, we sought to uncover genetic variants linked to T2D, CAD, and variations in gut microbiota and metabolites, aiming to shed light on the potential mechanisms connecting gut microbiota with DCAD. Our investigation uncovered a marked causal link between the presence of *Oxalobacter formigenes* and an increased incidence of both T2D and CAD. Specifically, a ten-unit genetic predisposition towards T2D was found to be associated with a 6.1% higher probability of an increase in the *Oxalobacteraceae* family's presence (β = 0.061, 95% CI = 0.002-0.119). In a parallel finding, an augmented presence of *Oxalobacter* was related to an 8.2% heightened genetic likelihood of CAD (β = 0.082, 95% CI = 0.026-0.137). This evidence indicates a critical pathway by which T2D can potentially raise the risk of CAD via alterations in gut microbiota. Additionally, our analyses reveal a connection between CAD risk and *Methanobacteria*, thus providing fresh perspectives on the roles of TMAO and carnitine in the etiology of CAD. The data also suggest a direct causal relationship between increased levels of certain metabolites — *proline*, *lysophosphatidylcholine*, *asparagine*, and *salicylurate* — and the prevalence of both T2D and CAD. Sensitivity assessments reinforce the notion that changes in *Oxalobacter formigenes* could pose a risk for DCAD. There is also evidence to suggest that DCAD may, in turn, affect the gut microbiota's makeup. Notably, a surge in serum TMAO levels in individuals with CAD, coinciding with a reduced presence of methanogens, has been identified as a potentially significant factor for future examination.

## Introduction

The diverse bacterial population within the human gut, numbering in the billions, plays a critical role in regulating host health and physiological functions 1. This microbial community is especially significant in the development and progression of various diseases, including cardiovascular maladies, metabolic disorders, neurogenic conditions, and immune system responses, with a particular impact on type 2 diabetes mellitus (T2D) and coronary artery disease (CAD) 2, 3. The imbalance of gut microbiota, known as dysbiosis, is increasingly acknowledged as a key contributor to metabolic imbalances, leading to persistent low-grade inflammation and oxidative stress, which are characteristic of T2D and its related health issues. Furthermore, the gut microbiota is known to participate actively in critical metabolic processes, contributing to the emergence of CAD by affecting inflammatory pathways and oxidative stress mechanisms 4. The likelihood of developing cardiovascular conditions is influenced by a confluence of factors, such as existing health conditions, lifestyle choices, and overall health 5, 6. Current research highlights the gut microbiota's significant role in mediating the risk and progression of CAD, particularly when it emerges as a secondary complication to diabetes 7.

Numerous studies have linked the gut microbiota to the development of T2D and CAD, highlighting the role of gut bacteria in the onset and progression of these conditions. It's well-documented that T2D significantly increases the risk of CAD, to an extent comparable to the risk associated with established heart diseases 8, 9. T2D-related issues such as hypertension and oxidative stress can lead to metabolic disturbances and impaired lipid metabolism, which in turn can cause both small and large vessel complications. These include a range of cardiovascular conditions that impact the arteries of various organs 10. Insulin resistance, a hallmark of T2D, is intricately connected to the composition of the gut microbiota 11. Specific bacterial species, including* Butyrivibrio crossotus*, *Eubacterium siraeum*,* Streptococcus mutans*, and *Eggerthella lenta*, play significant roles in regulating blood sugar levels by interacting with the gut's microbial ecosystem 12-14. Interestingly, shifts in the gut microbiome composition have been observed across different ethnic groups, including Asian and European populations, which have been shown to exhibit alterations in their gut microbiota in the context of T2D 15, 16.

Atherosclerotic cardiovascular conditions remain a leading contributor to disability and death among individuals with T2D. There is a growing body of evidence suggesting that the gut microbiota plays a crucial role in the development of atherosclerotic plaques 17, 18. The progression of atherosclerosis and CAD appears to be intricately linked to how the gut microbiota manages essential metabolic functions, notably affecting purine and lipid metabolism, as well as pathways related to oxidative stress and inflammation 5, 19.

The dynamic interplay between the gut microbiota's composition and diabetic coronary artery disease (DCAD) demands thorough investigation to establish direct causal links 20. It's increasingly critical to unravel how T2D enhances the susceptibility to CAD. Establishing causality in this domain is crucial not just for maintaining microbial equilibrium in the gut but also for developing strategies to prevent CAD.

Randomized controlled trials (RCTs) stand as the gold standard in epidemiological studies to determine causative relationships. However, their practical application can be restricted by logistical and ethical considerations. An alternative method, Mendelian randomization (MR), circumvents these limitations by employing genetic variants as proxies to draw causal inferences from observational data, thus minimizing confounder effects 21, 22. Leveraging the capabilities of MR, our research adopted a bidirectional two-sample MR method to substantiate the causal relationships between the gut microbiota and both T2D and CAD. Recent insights suggest that the interaction between gut microbiota and arterial health may play a role in how a lipid-rich diet contributes to atherosclerosis. Our MR examination of metabolites provides insights into their possible causative links with T2D and CAD 23.

## Materials and Methods

### Study Design

**Figure 1:**
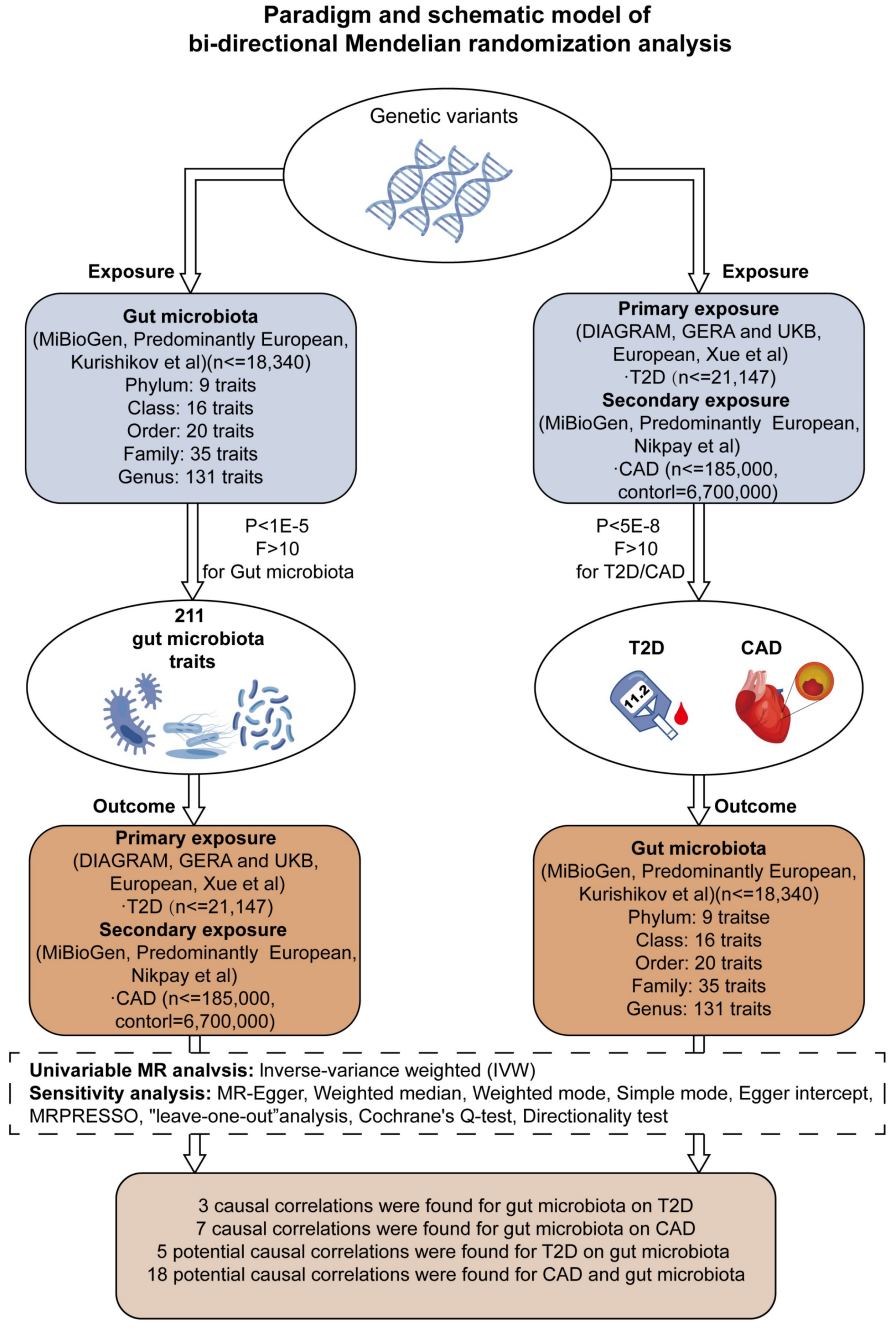
** Framework for Bidirectional MR Analysis.** This diagram details the methodological structure of our bidirectional Mendelian Randomization (MR) investigation, examining the cause-and-effect dynamics between gut microbiota and diseases such as type 2 diabetes (T2D) and coronary artery disease (CAD). Genetic data was primarily extracted from populations of European ancestry. The principal analysis method was inverse variance weighting (IVW), supplemented by sensitivity tests to ensure the reliability of the MR findings. After applying Bonferroni corrections, we identified significant causal links between three gut microbiota characteristics and T2D, and seven with CAD (P < 0.025, adjusted for two hypotheses). Notably, after adjustment for multiple testing (P < 2.36 × 10^-4, adjusted for 211 outcomes), no significant causal effect was observed between T2D/CAD and gut microbiota, although indicative causal links were noted.

Our research aimed to explore the genetic underpinnings of gut microbiota profiles and their influence on the incidence of T2D and CAD. By implementing a bidirectional two-sample Mendelian Randomization (MR) model, we assessed combined datasets from extensive genome-wide association studies (GWAS), with this process depicted in Figure 1 and elaborated upon in Supplementary Table S1. Furthermore, we conducted a one-way two-sample MR analysis to probe into the interactions between specific metabolites and the occurrence of T2D and CAD, along with their impact on the composition of the gut microbiota.

### Ethical Considerations and Methodological Conformance

This study incorporates data derived from GWAS databases that have undergone rigorous ethical scrutiny and received clearance for research utilization. The methodology adheres to the protocols established by Burgess and colleagues, and is in compliance with the recommendations outlined in the STROBE-MR guidelines for reporting observational research with Mendelian Randomization frameworks 24, 25.

### Data Acquisition and Genetic Marker Selection for T2D Analysis

For our investigation into T2D, we extracted data from a genome-wide association study (GWAS) by Xue et al. 26, which utilized samples from the DIAGRAM, GERA, and UKB cohorts. This pivotal study provided deeper insights into the genetic underpinnings of T2D and pinpointed potential gene loci for more in-depth functional studies. The findings from Xue et al. emphasized the significant impact of rare genetic variations on the risk associated with T2D. Our selection of genetic markers was based on a significance cut-off of 5×10^-8^, and we incorporated a linkage disequilibrium (LD) filter with an r^2^ value above 0.01 within a 5000 kb range. We calculated F-statistics for individual SNPs to confirm the strength of the genetic instruments, ensuring that each had an F-value well above 10, which is indicative of their reliability for use in MR analysis.

### Data Compilation for Coronary Artery Disease Investigation

For the assessment of CAD, we sourced information from an extensive GWAS meta-analysis undertaken by Nikpay et al. 27. This meta-analysis incorporated data from 48 distinct studies, totaling a cohort of 141,217 participants and close to 8.6 million SNPs. Instrumental variables selection for CAD mirrored the parameters set in the T2D analysis to maintain uniformity in our methodological approach.

### Genomic Insights into Gut Microbiota

For our analysis of gut microbiota, we utilized data from the mibioGen initiative 28, noted for being the most comprehensive GWAS collection to date. This repository includes data from 24 cohort studies, primarily involving individuals of European ancestry. It provides GWAS results for 211 different bacterial groups, spanning 9 phyla, 16 classes, 20 orders, 35 families, and 131 genera. The selection of instrumental variables for this aspect of the study was determined with a P-value threshold of less than 1×10^-5^, considering the relatively small pool of loci detected. We adopted the same linkage disequilibrium clumping strategy as in our analyses of T2D and CAD to ensure the genetic markers' validity 29.

### Compilation and Refinement of Metabolomic Data

We obtained our metabolomic data from a genome-wide association study by Rhee et al. 30, which analyzed blood metabolite profiles from 2,076 individuals of European descent participating in the Framingham Heart Study. This study focused on the relationship between gut microbiota and various host metabolites, taking into account numerous confounding factors such as age, gender, systolic blood pressure, antihypertensive drug use, body mass index (BMI), smoking status in diabetics, prevalence of cardiovascular diseases, and kidney function. These factors were adjusted to evaluate the correlations with 217 distinct metabolite concentrations in the dataset. For the subgroup analysis of metabolites, we set a P-value threshold of less than 1 × 10^-5^, consistent with the thresholds established in our prior analyses 31.

### Methodology for Statistical Analysis and Deduction of Causality

We utilized the inverse-variance weighted (IVW) method to assess causal links between 211 microbiome characteristics and both T2D and CAD. This assessment was conducted within the framework of a two-sample bidirectional MR, leveraging paired GWAS summary statistics. To address the concerns of multiple hypothesis testing and the possibility of horizontal pleiotropy - the scenario where genetic variants might affect disease outcomes via multiple pathways - our analysis incorporated supplementary MR methodologies, including MR-PRESSO, the weighted median approach, and MR Egger. We rigorously tested for the presence of multi-trait pleiotropy using the MR-PRESSO global tests and Cochrane's Q-statistics 32.

Causal relationships inferred from the gut microbiota's impact on T2D and CAD were quantified using beta coefficients, complete with 95% confidence intervals. We implemented the Bonferroni method for correcting multiple comparisons, considering causal effects as significant at P-values less than 0.025 for two specific outcomes and less than 2.36 × 10^-4^ for the broader 211 outcomes. P-values falling between 0.05 and the Bonferroni threshold were interpreted as suggestive of potential causal links.

The robustness of the MR findings was quantified using the mRnd1 online tool. All harmonized data pertinent to our study are accessible in Supplementary Material Data 1, while Supplementary Material Data 2 elaborates on the comprehensive outcomes of the bidirectional MR analysis, encompassing the gut microbiota, T2D, CAD, and related metabolites. Our MR analyses were conducted in the R statistical framework (version 4.2.2), using the TwoSampleMR (version 0.5.6) and MRPRESSO (version 1.0) packages. The TwoSampleMR package was instrumental in integrating exposure and outcome information, based on a thorough compilation of SNP data, including allele information, effect magnitudes, allele frequencies, and standard error metrics.

## Results

### SNP Selection for T2D and CAD Analysis

In our study, we rigorously filtered SNPs, excluding those within a 5000-kilobase pair range showing linkage disequilibrium (LD) with an r^2^ value exceeding 0.01, and also removed any duplicates. This stringent selection process identified 1,745 SNPs linked to T2D and 2,801 SNPs associated with CAD, each meeting a significance threshold of P < 1×10^-5^. Following this, our bidirectional two-sample MR analysis provided substantial evidence indicating an elevated risk of CAD in the context of T2D, as elaborated in Supplementary Table S2.

**Figure 2:**
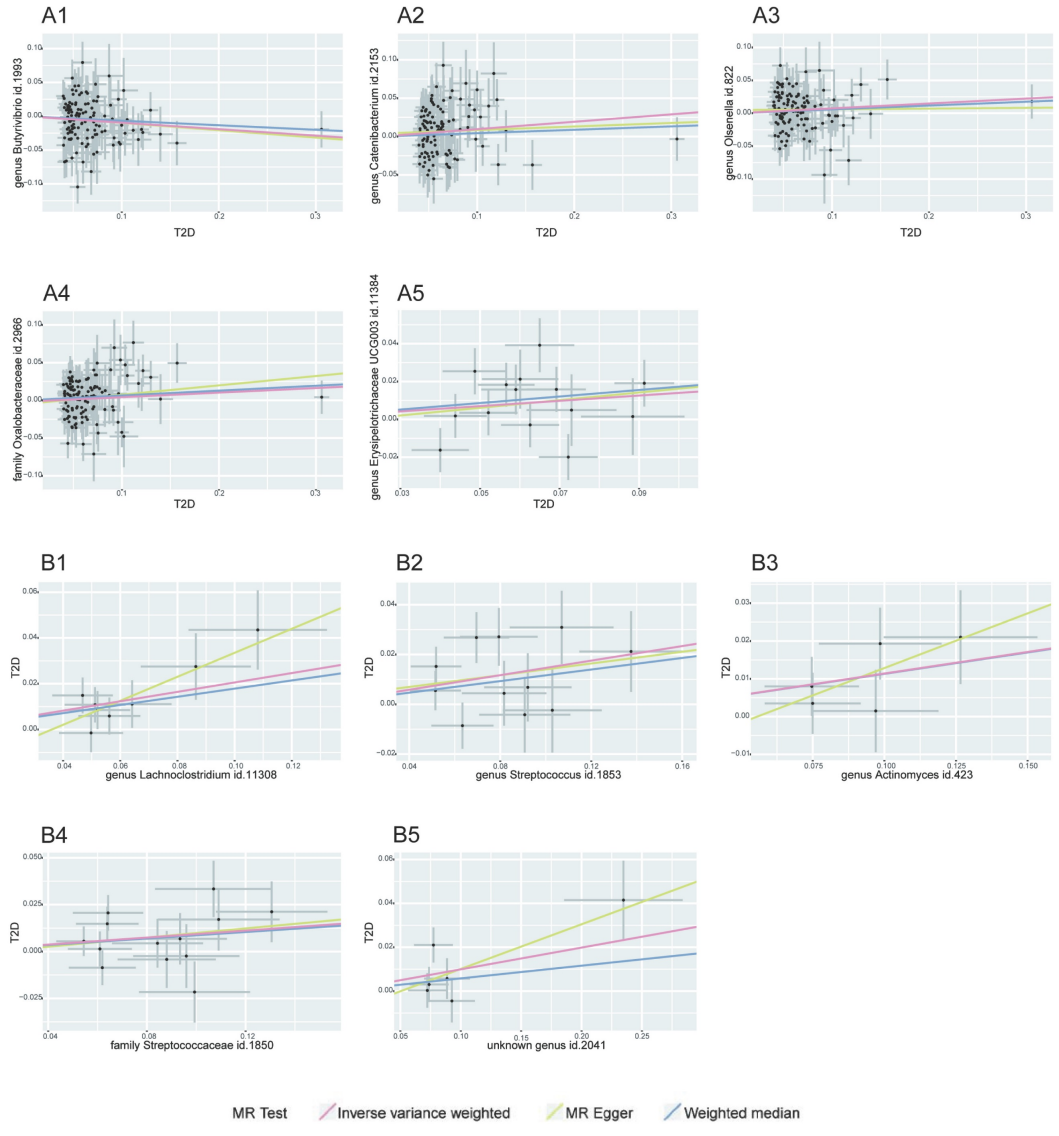

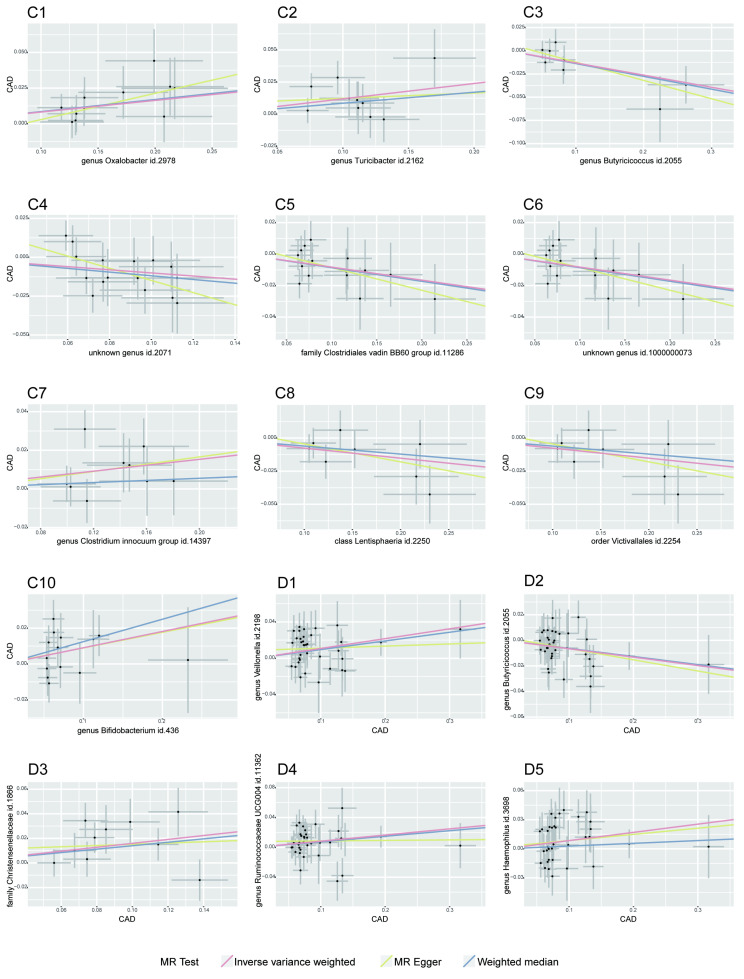

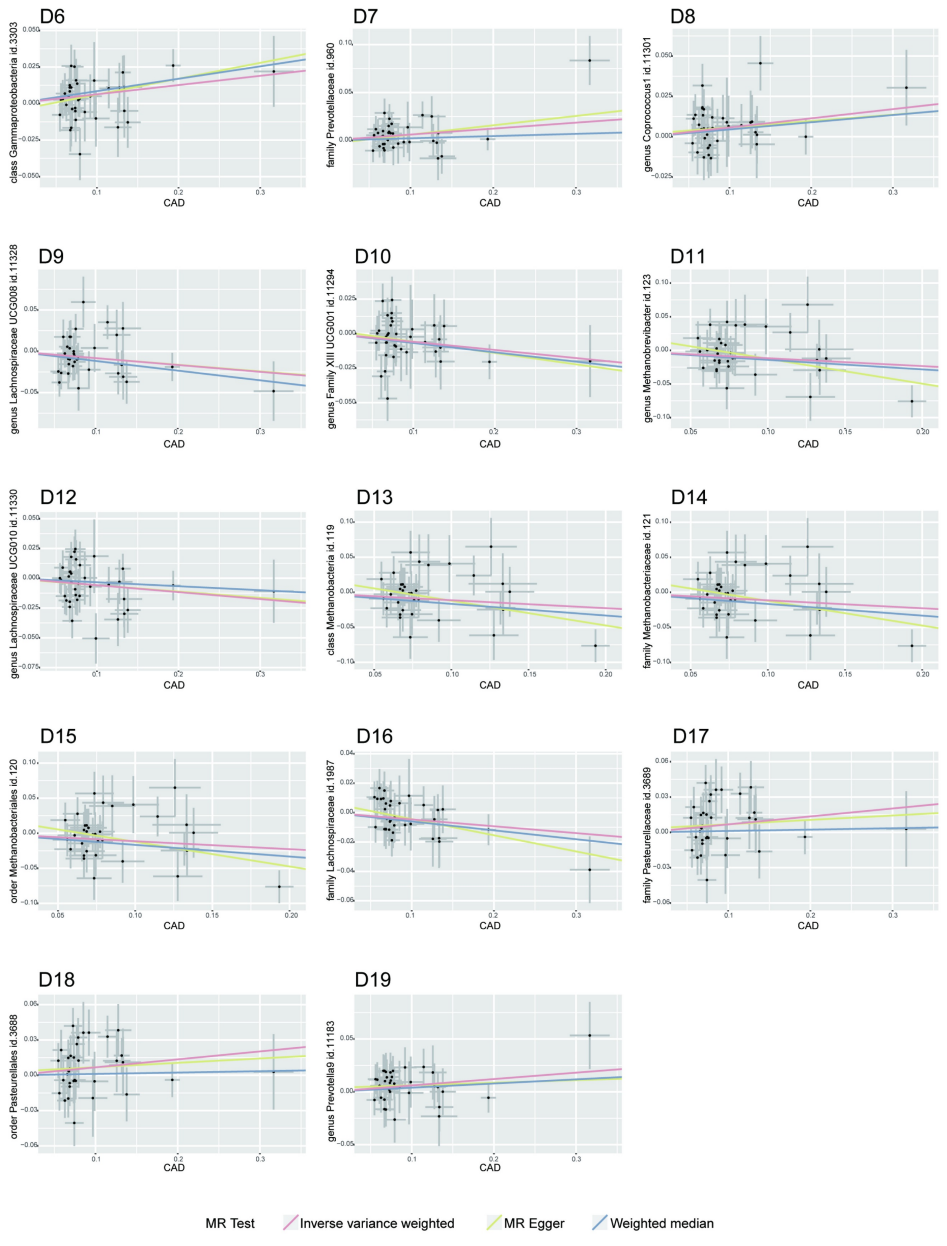
** MR Association Scatterplot for Gut Microbiota and Cardiometabolic Disorders.** The scatterplot features in panels A1-B5 illustrate the relationship between various gut microbiota traits and T2D. Panels C1-D17 display associations with CAD, revealing the range of genetic correlations investigated in this study.

Our MR analysis identified a total of 81 causal links, including those with potential associations where P < 0.05. This included five gut microbiota traits connected to T2D and ten to CAD, along with 16 metabolite traits associated with each condition. These findings were confirmed using MRPRESSO and leave-one-out analysis techniques, effectively ruling out instances of pleiotropy or heterogeneity. The reliability of these associations was further underscored by the F-statistics for the SNPs used in the MR analysis (see Tables 1-2, and Supplementary Tables S3-S4). A scatter plot in our report illustrates the trends and directionality of effects across different MR methodologies (see Figure 2).

In the bidirectional MR framework where T2D was considered as the exposure factor influencing CAD, a significant P-value of less than 0.05 was observed. While this result did not meet the criteria of the Cochran's Q test for heterogeneity, the existence of a P-value below 0.05 in a multiplicative random effects model pointed to a potential causal relationship between T2D and CAD, as noted in Supplementary Table S2.

### Impact of Gut Microbiota on T2D and CAD

In our investigation, we discerned nine distinct microbial taxa, spanning various taxonomic levels, that exhibit a positive causal relationship with both T2D and CAD. Regarding T2D, a genetic predisposition towards a greater abundance of the genera *Lachnoclostridium*, Streptococcus, Actinomyces, and the *Streptococcaceae* family was linked to a higher risk of the disease. Notably, a marked increase in *Lachnoclostridium* (β = 0.206, 95% CI = 0.095-0.316, P = 0.0002) was observed, indicating a significant rise in T2D risk (refer to Table 1). For CAD, elevated levels of *Oxalobacter, Turicibacter*, the *Clostridium innocuum* group, and *Bifidobacterium* were found to have a causative association with an increased risk, with Turicibacter showing a notable effect (β = 0.119, 95% CI = 0.076-0.163, P = 0.006), implying a considerable risk escalation for CAD (as shown in Table 2).

On the other hand, we identified that certain gut microbiota characteristics exhibit an inverse correlation with CAD risk. Specifically, the *Lentisphaeria* class, *Victivallales* order, *Clostridiales vadin BB60* family, and *Butyricicoccus* genus demonstrated a protective effect, as evidenced by beta coefficients ranging from -0.234 to -0.008, suggesting they may mitigate CAD progression.

While our data analysis didn't reveal any significant negative causal effects of gut microbiota on T2D, it did indicate that certain microbes are associated with a reduced CAD risk, pointing towards their potential protective influence against the condition, as detailed in Table 2.

### Effect of T2D and CAD on Gut Microbiota Dynamics

Our study explored the causal impact of T2D and CAD on the composition of gut microbiota, assessing causal links across 210 microbiotas for T2D and 211 for CAD. Four gut microbiotas exhibited positive causal links with T2D as a genetic factor, including the *genera Catenibacterium*, *Olsenella*, and *Erysipelotrichaceae* UCG-003, as well as the *Oxalobacteraceae* family. A genetic inclination towards T2D correlated with a heightened presence of these groups (*Catenibacterium* β = 0.096, 95% CI = 0.020-0.172, P = 0.013; *Olsenella* β = 0.074, 95% CI = 0.008-0.140, P = 0.027; *Erysipelotrichaceae* UCG-003 β = 0.140, 95% CI = 0.004-0.276, P = 0.043; *Oxalobacteraceae* β = 0.061, 95% CI = 0.002-0.119, P = 0.043), as indicated in Table 1. For CAD, an augmentation in several gut microbiota genera and families was noted, implying a possible connection post-Bonferroni adjustment (refer to Table 2).

In contrast, the *Butyrivibrio* genus showed a decrease in abundance with T2D, hinting at a possible protective role. Regarding CAD, a diminution in the abundance of certain gut microbiotas, such as *Butyricicoccus* and *Methanobacteriaceae*, was evident. Notably, the *Methanobacteria* genus displayed a significant reduction in abundance, suggesting a substantial protective influence against CAD.

To validate these conclusions, we conducted various sensitivity analyses, including MR-PRESSO, Cochrane's Q-test, and MR-Egger intercept tests. These procedures did not reveal any signs of heterogeneity or horizontal pleiotropy, thereby confirming the reliability of the identified causal relationships. Additionally, the F-values of the SNPs showing statistical significance consistently exceeded the threshold of 10, adding further credibility to our findings (as detailed in Supplementary Table S5).

### Metabolomic Influences on T2D and CAD

In conducting a MR study, coupled with Bonferroni adjustments for dual hypotheses (setting the significance threshold at P < 0.025), we identified a subset of 22 metabolites from a total of 217, which were genetically associated with a reduced risk of T2D. This selection encompassed a diverse array of metabolite classes, including but not limited to sphingomyelin (specifically *SM14_0*), selected amino acids, lysophosphatidylcholine (notably *LPC18_2*), triacylglycerol (specifically *TAG58_8*), certain adenosine derivatives, salicylurate, and glycerol. These metabolites demonstrated beta effect sizes in the range of -0.072 to -0.010, indicating their inverse relationship with T2D risk. In contrast, an increase in specific metabolites such as taurocholate, phosphatidylcholine (particularly *PC36_1*), and suberic acid was found to be genetically correlated with an elevated risk of T2D, with beta effect sizes ranging from 0.011 to 0.067.

### Metabolite-Gut Microbiota Interactions and CAD

In an analysis utilizing unidirectional MR, refined through Bonferroni adjustments (threshold set at P < 2.36 × 10^-4^), we were able to pinpoint four metabolites exhibiting causative links with both T2D and CAD. This assessment uncovered a negative causal association between proline levels and the presence of *Eubacterium xylanophilum* (yielding a beta coefficient of -0.038, within a 95% confidence interval of -0.058 to -0.019, and a P-value of 1.18×10^-4^). Furthermore, *LPC18_2* demonstrated a causal relationship with alterations in four distinct gut microbiota taxa. Significantly, an inverse correlation was observed between asparagine and the genus *Desulfovibrio* (beta coefficient of -0.059, 95% CI between -0.090 and -0.028, P = 1.80×10^-4^), while the Bacteroidales S24-7 group showed a positive correlation. Additionally, *salicylurate* was identified as having causative connections with both the *Christensenellaceae* family and the genus Coprococcus1, as detailed in Supplementary Data 2.

### Metabolite Associations with CAD

**Figure 3:**
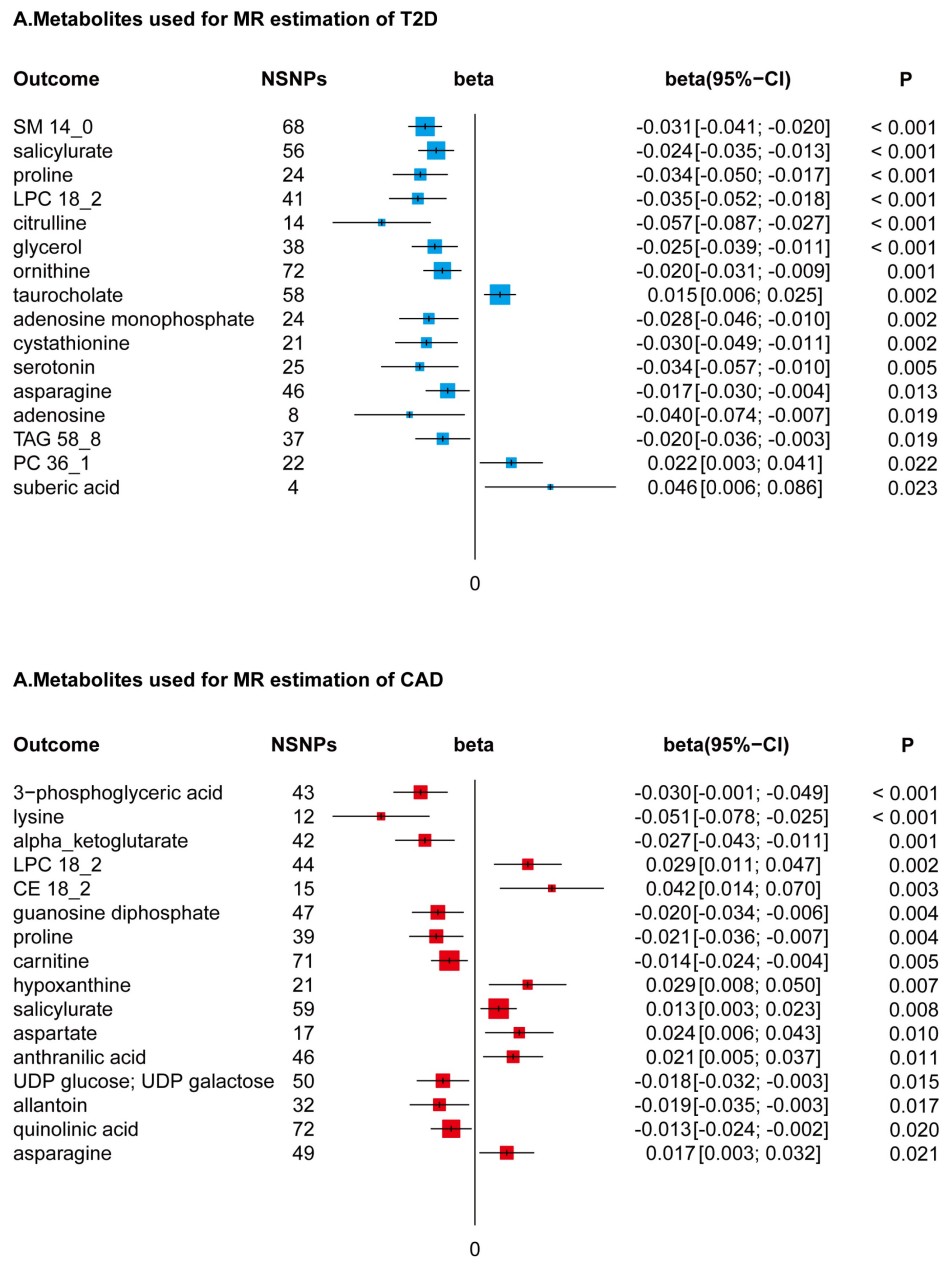
** Forest Plots of MR-Derived Causal Estimates.** Displayed here are the results from inverse variance-weighted MR analyses, examining the causal effects of different metabolites on T2D and CAD. Beta coefficients, along with 95% confidence intervals (CI), are shown, illustrating the variation in disease risk associated with each 10-unit increase in metabolite concentration. Analyzed metabolites include sphingomyelin (SM), lysophosphatidylcholine (LPC), triacylglycerol (TAG), phosphatidylcholine (PC), and cholesterol ester (CE).

Utilizing a directional two-sample MR approach, followed by a Bonferroni correction accommodating dual hypotheses (establishing a significance threshold at P < 0.025), our analysis discerned associations of 16 metabolites with CAD. Within this group, seven metabolites, notably* LPC18_2* and asparagine, were found to be genetically correlated with an increased predisposition to CAD. This correlation was quantified with beta effects spanning from 0.008 to 0.057. In contrast, a set of nine metabolites, which included amino acids like lysine and proline, exhibited a negative genetic association with CAD risk. The beta effect values for these metabolites varied from -0.067 to -0.007, as depicted in Figure 3.

## Discussion

In this investigation, we explored the reciprocal genetic relationships between the composition of the gut microbiota and the incidence of T2D and CAD. Our findings identified causal links of five gut microbiota characteristics with T2D, and ten with CAD. Conversely, our results suggest potential causal relationships of T2D with five gut microbiota types, and CAD with eighteen types. Additionally, we noted that certain metabolites, particularly those related to energy and lipids, exhibit causal connections with both T2D and CAD 33, 34.

The study identified five gut microbiota changes associated with T2D and ten with CAD. Of these, three microbiota types were causally linked to T2D, and seven to CAD. A notable causal association was observed between the increase in *Oxalobacteraceae* family abundance and T2D. In a surprising finding, a rise in the genus *Oxalobacter* was positively associated with an increased risk of CAD 35, 36. Noteworthy was the discovery that both *Turicibacter* and the *Clostridium innocuum* group shared the same single nucleotide polymorphism (SNP), rs4869133, suggesting its significance in the heightened risk of CAD linked to gut microbiota. Furthermore, the *Clostridiales vadin BB60* family, an unknown genus with the identifier *id.1000000073*, the *Lentisphaeria* class, and the *Victivallales* order all displayed identical SNPs in our final MR analysis. This genetic congruence might be attributed to the categorization of the unknown genus *id.1000000073* under the *Clostridiales vadin BB60* family, and a shared lineage between the *Victivallales* order and the *Lentisphaeria* class, indicating a limited range of genetic markers within these groups, as detailed in Table 3.

The anaerobic bacterium genus *Oxalobacter*, specialized in symbiosis and reliant solely on oxalic acid, was initially identified in the human gut and formally designated as *Oxalobacter formigenes* in 1985 37, 38. This bacterium has garnered significant attention in nephrolithiasis research due to correlations between heightened urinary oxalic acid excretion and the formation of oxalic acid kidney stones 39. Distinct variations in the gut microbiome have been noted in several studies comparing individuals with type 2 diabetes (T2D) and healthy controls. Key differences include a reduction in butyrate-producing gut microbiota, diminished levels of *Akkermansia muciniphila*, and an increased presence of pro-inflammatory bacterial species 40. Nonetheless, alterations in the abundance of *Lachnoclostridium*, *Streptococcus*, *Actinomyces*, and *Streptococcaceae* have been less frequently reported. Certain medications, like metformin, are known to modulate gut microbiota, thereby influencing insulin sensitivity and aiding in diabetes management. T2D may enhance the proliferation of *Oxalobacter formigenes* by inducing chronic intestinal inflammation and altering metabolic pathways related to oxalic acid processing 41, 42. This condition is characterized by heightened parasympathetic activity and local ATP release into the intestinal tract 43-45. The relatively unaffected colonization of *Oxalobacter formigenes* by other bacteria suggests a stable colonization characteristic of this genus 46. Research has examined various prevalent methods and conditions pertinent to probiotic strain production, particularly highlighting the resilience of the Group I *Oxalobacter strain OxCC13* in lyophilized form and when mixed in yogurt 47. Human consumption of *Oxalobacter* in these forms may offer preventive benefits against CAD, although the understanding of *Oxalobacter's* role in CAD remains incomplete 48. A gut microbiota-based diagnostic model suggests that increased gut colonization by* Oxalobacter formigenes* might elevate CAD risk 49. This aligns with our study findings, though the underlying mechanisms require further elucidation 50.

Recent studies focusing on the interplay between T2D and gut microbiota have observed a reduction in gut microbiota species that produce butyric acid in individuals with prediabetes, aligning with previous findings 40, 41. In the context of intestinal dysbiosis associated with T2D, metformin has been shown to enhance the production of butyric and propionic acids and improve patients' ability to break down amino acids. Additionally, metformin's role in modifying gut microbiota composition, potentially aiding in T2D prognosis through an increase in butyric acid-producing bacteria, has been highlighted 51, 52. Past research, encompassing both animal models and epidemiological studies, has underscored the bidirectional relationship between gut microbiota and host health in the context of atherosclerotic cardiovascular disease. Notably, bacterial presence in atherosclerotic plaques has been documented 53-55. The gut microbiota's influence on the metabolism of short-chain fatty acids (SCFAs), including *Prevotellaceae*, *Clostridium*, and *Anaerostipes*, has been linked to CAD, echoing findings from this study 56. A significant observation is the decreased abundance of methanogens in individuals susceptible to CAD. Certain methanogens are known to convert Trimethylamine (TMA) into a less harmful derivative, trimethylamine N-oxide (TMAO), thus reducing TMAO production 57. In our study, though the P-value in the IVW method for TMAO was less than 0.05, it did not pass sensitivity analyses, suggesting a potential connection between altered gut microbiota in coronary atherosclerosis patients and increased TMAO levels due to impaired metabolism.

For individuals with DCAD, long-term medication complicates the reliability of isolated gut microbiota observations. This study suggests that intestinal bacteria play a regulatory role in the development of both T2D and CAD, with implications for both elevated and reduced risk. The discovery of certain gut flora causally linked to diabetes and coronary heart disease, previously unreported, opens up new avenues for therapeutic strategies and potential targets.

The composition and activity of the gut microbiome, influenced by dietary and environmental factors, play a crucial role in the abundance and utilization of various metabolites 58. Metabolomics research has linked bile acids, branched-chain amino acids (BCAAs), and by-products of intracellular fatty acid oxidation to diabetes, glycemic control, and insulin resistance. Despite some studies indicating a correlation between TMAO levels and an increased risk of major cardiovascular events, including CAD, other studies have not found a significant relationship between circulating TMAO concentrations and cardiovascular outcomes 59-62. In our research, TMAO did not exhibit a notable association with CAD risk. However, we observed a positive correlation between certain lipid metabolism markers, such as phosphatidylcholine and cholesterol, including lysophosphatidylcholine (*LPC18_2*) and cholesterol ester (*CE18_2*), and CAD risk, underlining the strong connection between lipid metabolism and CAD 63-65. Animal studies have shown that rats on a carnitine-rich diet experienced a reduction in aortic lesion size, irrespective of increased blood TMAO levels, hinting at a possible protective role of carnitine against atherosclerosis 66, 67. This finding aligns with the results from our MR analysis, reinforcing the potential significance of carnitine in atherosclerosis prevention 68.

When evaluating the findings of this research, certain limitations must be acknowledged. Firstly, despite utilizing the most comprehensive genome-wide association study (GWAS) database currently available for gut microbiota and metabolites, the limited number of single nucleotide polymorphisms (SNPs) reaching genome-wide significance might have led to the use of weaker instrumental variables. To mitigate this, we expanded the inclusion criteria for SNPs to a statistical threshold of P < 10^-5^, allowing for a broader SNP inclusion. Additionally, to ascertain that these SNPs were not weak instrumental variables, they were evaluated using F statistics, ensuring values greater than 10. Secondly, given the extensive number of base pairs in the genome-wide analysis, it's challenging to completely rule out the presence of polymorphisms. Moreover, the biological implications of the selected SNPs have not been comprehensively explored. However, in our study, no horizontal pleiotropy was identified, as confirmed by the application of methods like MRPRESSO and MR Egger. Thirdly, our MR analysis was predicated on the assumption of a linear relationship between the variables of interest, hence the possibility of non-linear interactions between the exposure and outcome cannot be entirely dismissed. Finally, the metabolite database employed in our study was subject to preliminary screening. This limitation meant that a comprehensive two-way MR analysis was not feasible. Future research, ideally with more complete GWAS data, will be necessary to corroborate and expand upon our findings.

## Conclusion

The MR study conducted in our research provides insights into both the positive and negative causal effects of gut microbiota composition and metabolite levels on the occurrence of T2D and coronary artery disease (CAD). Our data supports the notion that the bacterial species *Oxalobacter formigenes* could be a contributory factor in CAD, particularly among individuals with diabetes. This study highlights a noteworthy link between the *Methanobacteria* class and CAD risk, paving the way for further exploration into the roles of TMAO and the protective potential of carnitine in the development of CAD. The findings present a new viewpoint on the influence of gut microbiota in the pathogenesis of CAD, providing valuable insights that could guide therapeutic approaches and the management of CAD in patients with T2D.

## Supplementary Material

Supplementary figures and tables.

Supplementary data 1.

Supplementary data 2.

## Figures and Tables

**Table 1:** Bidirectional MR Results of Type 2 diabetes and gut microbiota

Level	Exposure	Outcome	Method	NSNP	Beta(95%CI)	P	Directional pleiotropy		Cochrane's Q-statistic (P)	Steiger P
							Egger intercept (P)	MRPRESSO RSSobs (P)		
T2D on Gut microbiota										
Genus	T2D	Butyrivibrio	MR Egger	124	-0.108(-0.278,0.061)	0.212	0.001 (0.870)	151.361 (0.115)	143.251 (0.102)	6.51E-211
			Weighted median	124	-0.067(-0.195,0.061)	0.305				
			IVW	124	-0.096(-0.169,-0.022)	0.011				
Genus	T2D	Catenibacterium	MR Egger	114	0.046(-0.127,0.22)	0.603	0.004 (0.530)	126.627 (0.378)	121.413 (0.277)	1.51E-202
			Weighted median	114	0.044(-0.096,0.184)	0.537				
			IVW	114	0.096(0.02,0.172)	0.013				
Genus	T2D	Olsenella	MR Egger	124	0.011(-0.14,0.162)	0.886	0.005 (0.363)	135.497 (0.416)	122.308 (0.501)	5.77E-220
			Weighted median	124	0.058(-0.072,0.188)	0.379				
			IVW	124	0.074(0.008,0.14)	0.027				
Family	T2D	Oxalobacteraceae	MR Egger	125	0.124(-0.011,0.258)	0.073	-0.005 (0.307)	154.722 (0.106)	136.319 (0.212)	1.47E-212
			Weighted median	125	0.065(-0.037,0.167)	0.215				
			IVW	125	0.061(0.002,0.119)	0.043				
Genus	T2D	Erysipelotrichaceae UCG003	MR Egger	14	0.203(-0.418,0.823)	0.534	-0.004 (0.842)	23.752 (0.097)	20.351 (0.087)	2.09E-15
			Weighted median	14	0.173(0.011,0.334)	0.036				
			IVW	14	0.14(0.004,0.276)	0.043				
Gut microbiota on T2D										
Genus	Lachnoclostridium	T2D	MR Egger	8	0.524(0.044,1.005)	0.076	-0.019(0.230)	6.420(0.706)	4.971(0.664)	1.16E-23
			Weighted median	8	0.179(0.03,0.328)	0.019				
			IVW	8	0.206(0.095,0.316)	0.000				
Genus	Streptococcus	T2D	MR Egger	11	0.118(-0.239,0.474)	0.533	0.002(0.874)	19.848(0.147)	13.161(0.215)	4.19E-37
			Weighted median	11	0.116(-0.013,0.245)	0.077				
			IVW	11	0.146(0.046,0.246)	0.004				
Genus	Actinomyces	T2D	MR Egger	5	0.289(-0.185,0.763)	0.318	-0.016(0.514)	3.149(0.837)	2.163(0.706)	4.13E-18
			Weighted median	5	0.113(-0.008,0.234)	0.067				
			IVW	5	0.114(0.023,0.205)	0.014				
Family	Streptococcaceae	T2D	MR Egger	13	0.122(-0.218,0.462)	0.497	-0.002(0.867)	18.677(0.269)	13.621(0.326)	1.25E-44
			Weighted median	13	0.087(-0.029,0.203)	0.143				
			IVW	13	0.093(0.006,0.18)	0.035				
Genus	unknown genus id.2041	T2D	MR Egger	6	0.204(-0.072,0.48)	0.222	-0.010(0.472)	10.065(0.311)	6.910(0.227)	8.97E-19
			Weighted median	6	0.058(-0.056,0.172)	0.319				
			IVW	6	0.099(0.006,0.192)	0.037				

MR, mendelian randomization; T2D, Type 2 diabetes; IVW, inverse variance weighted; NSNPs, number of single nucleotide polymorphisms; beta, mendelian randomization effect estimate

**Table 2:** Bidirectional MR Results of Coronary artery disease and gut microbiota

Level	Exposure	Outcome	Method	NSNP	Beta(95%CI)	P	Directional pleiotropy		Cochrane's Q-statistic (P)	Steiger P
							Egger intercept (P)	MRPRESSO RSSobs (P)		
**Gut Microbiota on CAD**										
Genus	Oxalobacter	CAD	MR Egger	11	0.184(-0.075,0.444)	0.197	-0.016(0.447)	12.740(0.496)	4.155(0.940)	1.30E-36
			Weighted median	11	0.085(0.013,0.156)	0.020				
			IVW	11	0.082(0.026,0.137)	0.004				
Genus	Turicibacter	CAD	MR Egger	10	0.042 (-0.143, 0.226)	0.827	0.008(0.676)	14.681(0.478)	7.201(0.616)	5.77E-40
			Weighted median	10	0.085 (0.029, 0.142)	0.132				
			IVW	10	0.119 (0.076, 0.163)	0.006				
Genus	Butyricicoccus	CAD	MR Egger	8	-0.197(-0.381, -0.014)	0.080	0.007(0.426)	10.029(0.494)	4.227(0.753)	5.00E-24
			Weighted median	8	-0.138(-0.279,0.003)	0.056				
			IVW	8	-0.131(-0.234, -0.028)	0.012				
Genus	unknown genus id.2071	CAD	MR Egger	16	-0.392(-0.764, -0.02)	0.058	0.024(0.139)	28.083(0.141)	13.176(0.589)	9.07E-51
			Weighted median	16	-0.119(-0.23, -0.008)	0.036				
			IVW	16	-0.101(-0.18, -0.021)	0.013				
Family	Clostridiales vadin BB60 group	CAD	MR Egger	15	-0.144(-0.345,0.057)	0.184	0.006(0.536)	9.383(0.945)	7.743(0.902)	2.85E-50
			Weighted median	15	-0.086(-0.177,0.004)	0.062				
			IVW	15	-0.083(-0.153, -0.013)	0.021				
Genus	unknown genus id.1000000073	CAD	MR Egger	15	-0.144(-0.345,0.057)	0.184	0.006(0.536)	9.383(0.940)	7.743(0.902)	2.85E-50
			Weighted median	15	-0.086(-0.175,0.003)	0.057				
			IVW	15	-0.083(-0.153, -0.013)	0.021				
Genus	Clostridium innocuum group	CAD	MR Egger	9	0.094(-0.262,0.45)	0.620	-0.002(0.924)	14.581(0.30)9	8.562(0.381)	1.10E-28
			Weighted median	9	0.028(-0.06,0.115)	0.537				
			IVW	9	0.077(0.011,0.142)	0.022				
Class	Lentisphaeria	CAD	MR Egger	8	-0.135(-0.371,0.1)	0.303	0.009(0.625)	5.244(0.908)	3.979(0.782)	3.79E-29
			Weighted median	8	-0.061(-0.152,0.031)	0.194				
			IVW	8	-0.076(-0.144, -0.008)	0.028				
Order	Victivallales	CAD	MR Egger	8	-0.135(-0.371,0.1)	0.303	0.009(0.625)	5.244(0.897)	3.979(0.782)	3.79E-29
			Weighted median	8	-0.061(-0.145,0.024)	0.160				
			IVW	8	-0.076(-0.144, -0.008)	0.028				
Genus	Bifidobacterium	CAD	MR Egger	14	0.087(-0.147,0.321)	0.482	0.000(0.972)	18.136(0.464)	11.777(0.546)	3.50E-58
			Weighted median	14	0.125(0.014,0.235)	0.027				
			IVW	14	0.091(0.008,0.173)	0.031				
**CAD on Gut Microbiota**										
Genus	CAD	Veillonella	MR Egger	36	0.023(-0.13,0.177)	0.77	0.009 (0.243)	34.134 (0.812)	29.682 (0.722)	1.80E-85
			Weighted median	36	0.095(-0.001,0.192)	0.052				
			IVW	36	0.108(0.045,0.171)	0.001				
Genus	CAD	Butyricicoccus	MR Egger	36	-0.088(-0.199,0.024)	0.134	0.002 (0.678)	29.168 (0.934)	23.637 (0.928)	8.15E-91
			Weighted median	36	-0.063(-0.131,0.005)	0.069				
			IVW	36	-0.066(-0.112, -0.019)	0.005				
Family	CAD	Christensenellaceae	MR Egger	10	0.05(-0.367,0.468)	0.819	0.01 (0.609)	17.859 (0.182)	11.086 (0.27)	4.40E-14
			Weighted median	10	0.14(-0.005,0.285)	0.059				
			IVW	10	0.159(0.046,0.272)	0.006				
Genus	CAD	Ruminococcaceae UCG004	MR Egger	36	0.007(-0.142,0.156)	0.928	0.008 (0.281)	34.257 (0.784)	30.473 (0.686)	5.60E-87
			Weighted median	36	0.074(-0.023,0.171)	0.134				
			IVW	36	0.083(0.021,0.145)	0.009				
Genus	CAD	Haemophilus	MR Egger	36	0.064(-0.095,0.222)	0.438	0.002 (0.774)	42.824 (0.453)	35.787 (0.431)	7.65E-84
			Weighted median	36	0.028(-0.071,0.128)	0.575				
			IVW	36	0.085(0.02,0.149)	0.010				
Class	CAD	Gammaproteobacteria	MR Egger	36	0.109(-0.008,0.225)	0.076	-0.005 (0.408)	38.906 (0.609)	28.717 (0.764)	3.77E-86
			Weighted median	36	0.085(0.016,0.154)	0.016				
			IVW	36	0.063(0.015,0.111)	0.010				
Family	CAD	Prevotellaceae	MR Egger	36	0.098(-0.024,0.219)	0.124	-0.004 (0.533)	31.991 (0.865)	26.465 (0.85)	9.13E-91
			Weighted median	36	0.023(-0.055,0.101)	0.563				
			IVW	36	0.062(0.012,0.112)	0.015				
Genus	CAD	Coprococcus1	MR Egger	36	0.039(-0.074,0.153)	0.5	0.002 (0.74)	33.98 (0.803)	26.142 (0.86)	1.19E-89
			Weighted median	36	0.045(-0.024,0.114)	0.203				
			IVW	36	0.057(0.01,0.104)	0.017				
Genus	CAD	Lachnospiraceae UCG008	MR Egger	35	-0.08(-0.256,0.096)	0.38	0(0.973)	42.248 (0.401)	33.612 (0.486)	1.43E-83
			Weighted median	35	-0.117(-0.223, -0.011)	0.03				
			IVW	35	-0.083(-0.156, -0.01)	0.025				
Genus	CAD	Family XIII UCG001	MR Egger	36	-0.082(-0.211,0.046)	0.217	0.002 (0.699)	33.55 (0.819)	30.732 (0.674)	1.43E-89
			Weighted median	36	-0.068(-0.144,0.009)	0.082				
			IVW	36	-0.059(-0.113, -0.006)	0.03				
Genus	CAD	Methanobrevibacter	MR Egger	34	-0.37(-0.667, -0.073)	0.02	0.024 (0.083)	37.82 (0.596)	32.384 (0.498)	6.55E-78
			Weighted median	34	-0.141(-0.304,0.021)	0.088				
			IVW	34	-0.117(-0.224, -0.01)	0.032				
Genus	CAD	Lachnospiraceae UCG010	MR Egger	36	-0.053(-0.188,0.082)	0.447	-0.001 (0.934)	40.419 (0.522)	38.02 (0.333)	3.35E-85
			Weighted median	36	-0.033(-0.115,0.048)	0.42				
			IVW	36	-0.058(-0.113, -0.003)	0.038				
Class	CAD	Methanobacteria	MR Egger	34	-0.352(-0.647, -0.057)	0.026	0.023 (0.099)	40.407 (0.493)	35.488 (0.352)	1.44E-76
			Weighted median	34	-0.166(-0.337,0.005)	0.057				
			IVW	34	-0.114(-0.223, -0.004)	0.042				
Family	CAD	Methanobacteriaceae	MR Egger	34	-0.352(-0.647, -0.057)	0.026	0.023 (0.099)	40.407 (0.494)	35.488 (0.352)	1.44E-76
			Weighted median	34	-0.166(-0.332,0)	0.05				
			IVW	34	-0.114(-0.223, -0.004)	0.042				
Order	CAD	Methanobacteriales	MR Egger	34	-0.352(-0.647, -0.057)	0.026	0.023 (0.099)	40.407 (0.451)	35.488 (0.352)	1.44E-76
			Weighted median	34	-0.166(-0.331, -0.001)	0.048				
			IVW	34	-0.114(-0.223, -0.004)	0.042				
Family	CAD	Lachnospiraceae	MR Egger	36	-0.11(-0.219, -0.001)	0.055	0.007 (0.217)	25.906 (0.969)	21.433 (0.965)	3.95E-95
			Weighted median	36	-0.06(-0.125,0.004)	0.066				
			IVW	36	-0.046(-0.091, -0.002)	0.042				
Family	CAD	Pasteurellaceae	MR Egger	36	0.037(-0.127,0.201)	0.66	0.003 (0.692)	45.756 (0.349)	39.428 (0.278)	1.81E-83
			Weighted median	36	0.012(-0.089,0.113)	0.822				
			IVW	36	0.068(0.001,0.134)	0.047				
Order	CAD	Pasteurellales	MR Egger	36	0.037(-0.127,0.201)	0.66	0.003 (0.692)	45.756 (0.324)	39.428 (0.278)	1.81E-83
			Weighted median	36	0.012(-0.083,0.106)	0.81				
			IVW	36	0.068(0.001,0.134)	0.047				
Genus	CAD	Prevotella9	MR Egger	36	0.024(-0.123,0.172)	0.748	0.004 (0.604)	26.219 (0.979)	20.101 (0.979)	2.75E-95
			Weighted median	36	0.039(-0.048,0.127)	0.377				
			IVW	36	0.06(0,0.121)	0.05				

MR, mendelian randomization; CAD, Coronary artery disease; IVW, inverse variance weighted; NSNP, number of single nucleotide polymorphisms; beta, mendelian randomization effect estimate

**Table 3:** Particulars of SNPs used in MR analyses of gut microbiota

Exposure traits	SNPs	EA	OA	Beta	Se	samplesize	P-value	R^2^	F-statistic
Type 2 diabetes (P<1×10^-12^)	rs2296173	G	A	0.065	0.0087	62892	7.65773E-14	0.001	55.820
	rs340874	C	T	0.0626	0.0073	62892	8.40621E-18	0.001	73.536
	rs2972144	G	A	0.0913	0.0075	62892	2.55094E-34	0.002	148.190
	rs243019	C	T	0.0566	0.0071	62892	2.28981E-15	0.001	63.550
	rs780094	C	T	0.0692	0.0074	62892	5.15941E-21	0.001	87.448
	rs17334919	T	C	-0.1398	0.0128	62892	6.68652E-28	0.002	119.287
	rs13389219	T	C	-0.0722	0.0074	62892	2.1062E-22	0.002	95.194
	rs6808574	C	T	0.0552	0.0076	62892	4.38531E-13	0.001	52.753
	rs11708067	G	A	-0.0965	0.0086	62892	5.93335E-29	0.002	125.909
	rs6795735	T	C	-0.0558	0.0073	62892	1.63005E-14	0.001	58.428
	rs7651090	G	A	0.1204	0.0076	62892	3.8539E-57	0.004	250.972
	rs1899951	T	C	-0.1118	0.0109	62892	1.63682E-24	0.002	105.204
	rs1496653	G	A	-0.0769	0.0088	62892	2.57217E-18	0.001	76.364
	rs1801214	T	C	0.0903	0.0074	62892	5.51569E-34	0.002	148.906
	rs459193	G	A	0.0711	0.0083	62892	8.80846E-18	0.001	73.381
	rs7729395	T	C	0.1373	0.016	62892	1.10103E-17	0.001	73.638
	rs7756992	G	A	0.1297	0.0078	62892	5.99929E-62	0.004	276.497
	rs1063355	G	T	0.0709	0.0079	62892	3.71535E-19	0.001	80.545
	rs17168486	T	C	0.0742	0.0094	62892	2.17721E-15	0.001	62.309
	rs2191348	T	G	0.0652	0.0073	62892	3.44429E-19	0.001	79.772
	rs13234269	A	T	-0.0583	0.0078	62892	6.9775E-14	0.001	55.866
	rs849135	A	G	-0.0999	0.0072	62892	1.04112E-43	0.003	192.516
	rs3802177	A	G	-0.1217	0.008	62892	2.32113E-52	0.004	231.420
	rs516946	C	T	0.0824	0.0085	62892	3.15864E-22	0.001	93.976
	rs10974438	C	A	0.0591	0.0075	62892	3.01301E-15	0.001	62.094
	rs10811661	C	T	-0.1569	0.0098	62892	4.13238E-58	0.004	256.327
	rs2796441	A	G	-0.0715	0.0073	62892	1.962E-22	0.002	95.933
	rs1063192	A	G	0.0634	0.0073	62892	3.29837E-18	0.001	75.428
	rs4918796	C	T	0.0623	0.0086	62892	4.01328E-13	0.001	52.478
	rs7923866	T	C	-0.0972	0.0074	62892	9.33684E-40	0.003	172.532
	rs11257655	T	C	0.0737	0.0087	62892	1.96607E-17	0.001	71.762
	rs7903146	T	C	0.3059	0.0077	62892	1E-200	0.024	1578.256
	rs10830963	G	C	0.0909	0.008	62892	5.84655E-30	0.002	129.106
	rs1552224	C	A	-0.1034	0.0101	62892	8.63575E-25	0.002	104.809
	rs5215	T	C	-0.0678	0.0073	62892	2.08882E-20	0.001	86.261
	rs10842994	T	C	-0.0755	0.0091	62892	1.01508E-16	0.001	68.835
	rs2261181	T	C	0.0985	0.0118	62892	9.1791E-17	0.001	69.680
	rs825476	T	C	0.0524	0.0073	62892	6.80456E-13	0.001	51.525
	rs61953351	T	G	-0.07	0.0091	62892	1.97606E-14	0.001	59.172
	rs1359790	A	G	-0.0796	0.008	62892	2.79512E-23	0.002	99.003
	rs7177055	A	G	0.0647	0.0079	62892	2.746E-16	0.001	67.074
	rs7185735	G	A	0.1056	0.0073	62892	1.59001E-47	0.003	209.258
	rs77258096	A	C	-0.1171	0.0134	62892	1.7832E-18	0.001	76.367
	rs8068804	A	G	0.0587	0.0078	62892	4.41062E-14	0.001	56.635
	rs9894220	G	A	-0.0585	0.0079	62892	1.51705E-13	0.001	54.835
	rs8108269	G	T	0.0644	0.0079	62892	3.11387E-16	0.001	66.453
coronary artery disease (P<1×10^-10^)	rs67180937	G	T	0.078807	0.0110551	42457	1.01E-12	0.001	50.816
	rs7528419	G	A	-0.11453	0.011482	42457	1.97E-23	0.002	99.495
	rs9970807	T	C	-0.12575	0.016695	42457	5.00E-14	0.001	56.734
	rs115654617	A	C	0.137846	0.0158314	42457	3.12E-18	0.002	75.814
	rs12202017	G	A	-0.066813	0.0099612	42457	1.98E-11	0.001	44.988
	rs55730499	T	C	0.316641	0.0242403	42457	5.39E-39	0.004	170.631
	rs186696265	T	C	0.550351	0.0481949	42457	3.35E-30	0.003	130.400
	rs9349379	G	A	0.131836	0.0096527	42457	1.81E-42	0.004	186.539
	rs2107595	A	G	0.073415	0.0112951	42457	8.05E-11	0.001	42.246
	rs11556924	T	C	-0.072569	0.0110605	42457	5.34E-11	0.001	43.048
	rs2891168	G	A	0.193401	0.0091877	42457	2.29E-98	0.010	443.102
	rs2487928	A	G	0.062633	0.0095049	42457	4.41E-11	0.001	43.422
	rs1870634	G	T	0.075878	0.0097113	42457	5.55E-15	0.001	61.049
	rs1412444	T	C	0.066812	0.0096809	42457	5.15E-12	0.001	47.630
	rs2128739	C	A	-0.065565	0.0100568	42457	7.05E-11	0.001	42.503
	rs2681472	G	A	0.074114	0.0113331	42457	6.17E-11	0.001	42.766
	rs4468572	C	T	0.077234	0.0095277	42457	4.44E-16	0.002	65.711
	rs4420638	G	A	0.091906	0.0140977	42457	7.07E-11	0.001	42.500
	rs56289821	A	G	-0.13361	0.0170415	42457	4.44E-15	0.001	61.470
	rs28451064	A	G	0.127571	0.015952	42457	1.33E-15	0.002	63.955
genus Lachnoclostridium id.11308 (P<1×10^-5^)	rs12566975	T	C	-0.0468097	0.0105787	14306	9.57194E-06	0.001	19.580
	rs1528479	A	G	0.0497799	0.0111919	14306	9.63984E-06	0.001	19.783
	rs615997	T	C	0.0511752	0.0106491	14306	2.0268E-06	0.002	23.094
	rs62285313	A	G	0.0864203	0.0181565	14306	1.58332E-06	0.002	22.655
	rs1031599	T	G	0.078627	0.0175644	14306	6.31379E-06	0.001	20.039
	rs3821998	C	A	-0.0864066	0.0192519	14306	6.72048E-06	0.001	20.144
	rs4738679	A	G	0.0520267	0.011404	14306	4.41754E-06	0.001	20.813
	rs1997204	C	T	0.108075	0.0242022	14306	5.97077E-06	0.001	19.941
	rs62028349	G	C	0.0469989	0.0105971	14306	9.17044E-06	0.001	19.670
	rs72829893	G	T	0.117472	0.0268103	14306	5.57763E-06	0.001	19.198
	rs78068103	A	G	0.0886199	0.0194248	14306	3.66522E-06	0.001	20.814
	rs2385421	A	G	0.0746186	0.0180734	14306	7.13724E-06	0.001	17.046
	rs789029	C	T	-0.0641288	0.0137974	14306	3.75327E-06	0.002	21.603
	rs6112314	A	C	-0.0561715	0.0108174	14306	2.43215E-07	0.002	26.964
genus Streptococcus id.1853 (P<1×10^-5^)	rs11720390	G	A	0.107024	0.0228121	14306	3.59484E-06	0.002	22.011
	rs6806351	T	C	-0.0633829	0.0136647	14306	4.93867E-06	0.002	21.515
	rs57646748	G	A	-0.0907696	0.0200344	14306	5.47545E-06	0.001	20.527
	rs10028567	C	T	-0.0921167	0.0191881	14306	7.30348E-06	0.002	23.047
	rs395407	C	G	0.0792781	0.0173697	14306	4.36506E-06	0.001	20.832
	rs77558518	A	G	-0.103999	0.0229714	14306	4.70858E-06	0.001	20.497
	rs11764382	A	G	-0.0695345	0.0143671	14306	1.28632E-06	0.002	23.424
	rs17708276	A	G	-0.0793955	0.0170628	14306	3.04096E-06	0.002	21.652
	rs10448310	A	G	-0.0517935	0.0111324	14306	3.30704E-06	0.002	21.646
	rs71481756	T	G	0.0931048	0.0207949	14306	6.51478E-06	0.001	20.046
	rs7916711	A	G	0.102891	0.0217362	14306	0.000002717	0.002	22.407
	rs1918540	A	G	-0.059639	0.0128148	14306	2.44068E-06	0.002	21.659
	rs11110281	T	C	-0.137519	0.0227398	14306	2.58315E-09	0.003	36.572
	rs2370083	G	T	-0.0816836	0.0185851	14306	9.75237E-06	0.001	19.317
	rs72739637	A	G	0.0959942	0.0193213	14306	1.03307E-06	0.002	24.684
	rs6563952	C	G	-0.0827344	0.0180035	14306	5.8213E-06	0.001	21.118
	rs4968759	A	G	-0.0515109	0.0112068	14306	3.7812E-06	0.001	21.127
	rs9903102	C	A	-0.0709483	0.0155275	14306	4.17994E-06	0.001	20.878
genus Actinomyces id.423 (P<1×10^-5^)	rs71315246	A	G	-0.0969809	0.021925	14306	9.82969E-06	0.001	19.566
	rs34583783	G	T	0.126596	0.0268461	14306	4.48528E-06	0.002	22.237
	rs4073240	G	A	0.0749687	0.0167368	14306	7.94273E-06	0.001	20.064
	rs35011108	A	G	0.232634	0.0512044	14306	6.33826E-06	0.001	20.641
	rs4146653	G	A	0.0985224	0.0214182	14306	4.49645E-06	0.001	21.159
	rs10787984	G	C	0.094316	0.0213513	14306	9.62299E-06	0.001	19.513
	rs7915461	C	T	-0.18776	0.0401636	14306	5.91984E-06	0.002	21.855
	rs2715439	T	C	-0.0746684	0.0164822	14306	6.27004E-06	0.001	20.523
family Streptococcaceae id.1850 (P<1×10^-5^)	rs77968078	G	A	-0.0993013	0.0224788	14306	7.93341E-06	0.001	19.515
	rs76717940	T	A	0.150606	0.0334079	14306	3.08937E-06	0.001	20.323
	rs6806351	T	C	-0.0619209	0.0135744	14306	6.93793E-06	0.001	20.808
	rs10028567	C	T	-0.0934027	0.0190343	14306	3.72495E-06	0.002	24.079
	rs57646748	G	A	-0.088021	0.019876	14306	7.88352E-06	0.001	19.612
	rs395407	C	G	0.0826855	0.0172536	14306	1.32559E-06	0.002	22.967
	rs77558518	A	G	-0.104239	0.022806	14306	3.72195E-06	0.001	20.891
	rs957755	T	G	-0.0642449	0.0142702	14306	7.41515E-06	0.001	20.268
	rs2952251	G	A	0.0639298	0.0126525	14306	3.72237E-07	0.002	25.530
	rs28718126	A	G	0.109069	0.0246868	14306	9.41044E-06	0.001	19.520
	rs7916711	A	G	0.0959639	0.021545	14306	6.32732E-06	0.001	19.839
	rs16950051	A	G	0.107008	0.0236973	14306	5.33814E-06	0.001	20.391
	rs11110281	T	C	-0.130554	0.0225943	14306	1.40136E-08	0.002	33.387
	rs2370083	G	T	-0.0842751	0.0184509	14306	4.25667E-06	0.001	20.862
	rs72739637	A	G	0.0927983	0.0192021	14306	1.82163E-06	0.002	23.355
	rs6563952	C	G	-0.0801931	0.0178576	14306	8.70583E-06	0.001	20.166
	rs35344081	G	A	0.0609349	0.0129703	14306	2.63846E-06	0.002	22.072
	rs9903102	C	A	-0.0693015	0.0154096	14306	4.91802E-06	0.001	20.226
	rs4968759	A	G	-0.0544035	0.0111271	14306	8.91887E-07	0.002	23.905
unknown genus id.2041 (P<1×10^-5^)	rs1032598	G	A	-0.0886426	0.0189039	14306	4.07587E-06	0.002	21.988
	rs16843660	A	G	0.234697	0.04907	14306	1.75344E-06	0.002	22.876
	rs11941716	A	G	0.101243	0.0224414	14306	9.0663E-06	0.001	20.353
	rs249459	A	G	0.0737341	0.0165018	14306	8.12307E-06	0.001	19.965
	rs553072	G	A	0.109193	0.0230198	14306	3.69097E-06	0.002	22.500
	rs1962916	G	A	-0.0737876	0.0162232	14306	6.13847E-06	0.001	20.687
	rs35703006	G	T	0.0926669	0.0190779	14306	9.00762E-07	0.002	23.593
	rs921383	G	A	0.0723153	0.0159706	14306	7.72894E-06	0.001	20.503
	rs2651663	A	G	-0.0762556	0.0168635	14306	5.64144E-06	0.001	20.448
	rs2336448	T	C	0.0773742	0.0160883	14306	1.42899E-06	0.002	23.130
	rs7187855	A	C	0.199941	0.0418308	14306	2.20602E-06	0.002	22.846
	rs6514318	T	C	0.128198	0.0281765	14306	5.37675E-06	0.001	20.701
genus Oxalobacter id.2978 (P<1×10^-5^)	rs4428215	G	A	0.130293	0.0242237	14306	7.51069E-08	0.002	28.931
	rs36057338	G	T	0.207847	0.0421439	14306	8.79812E-07	0.002	24.323
	rs1569853	T	C	-0.138078	0.0296981	14306	3.64502E-06	0.002	21.617
	rs6993398	G	A	0.127217	0.0278855	14306	7.12771E-06	0.001	20.813
	rs10464997	G	A	0.137691	0.0294804	14306	3.29754E-06	0.002	21.814
	rs12002250	A	C	0.217122	0.0466317	14306	1.41504E-06	0.002	21.679
	rs736744	T	C	-0.117882	0.0211262	14306	2.57472E-08	0.002	31.135
	rs3862635	C	T	-0.172142	0.0394026	14306	9.18692E-06	0.001	19.086
	rs11108500	A	G	-0.199099	0.0427327	14306	3.74283E-06	0.002	21.708
	rs111966731	T	C	0.213114	0.047162	14306	7.29861E-06	0.001	20.419
	rs6071435	T	A	-0.105512	0.021489	14306	1.07431E-06	0.002	24.109
	rs6000536	C	T	-0.130992	0.0253804	14306	2.06054E-07	0.002	26.637
genus Turicibacter id.2162 (P<1×10^-5^)	rs149744580	A	G	0.169883	0.0315478	14306	7.00971E-08	0.002	28.998
	rs4869133	G	A	0.131186	0.027197	14306	2.5537E-06	0.002	23.267
	rs2221441	G	C	0.0710364	0.015343	14306	3.45669E-06	0.001	21.436
	rs3734633	G	A	-0.120957	0.02683	14306	5.31912E-06	0.001	20.325
	rs55756211	T	C	-0.115115	0.0240708	14306	2.8053E-06	0.002	22.871
	rs2952020	A	G	0.0759019	0.0165764	14306	5.63313E-06	0.001	20.966
	rs61265175	G	C	-0.0858591	0.0185778	14306	4.13676E-06	0.001	21.359
	rs11054680	T	C	-0.104751	0.0226997	14306	2.30978E-06	0.001	21.295
	rs4247078	G	C	-0.0710377	0.0155221	14306	5.46072E-06	0.001	20.945
	rs11649454	G	C	0.0950891	0.0203433	14306	3.26625E-06	0.002	21.848
	rs7199484	G	A	-0.0731428	0.0160172	14306	5.7666E-06	0.001	20.853
	rs12603364	T	C	0.110861	0.0225598	14306	8.66603E-07	0.002	24.148
	rs11666533	C	T	-0.111689	0.0248436	14306	7.37106E-06	0.001	20.211
	rs2834977	T	C	-0.0959995	0.0208261	14306	3.95585E-06	0.001	21.248
genus Butyricicoccus id.2055 (P<1×10^-5^)	rs12034718	G	A	-0.0701199	0.0158213	14306	9.57679E-06	0.001	19.643
	rs10084203	G	A	-0.0549699	0.0123563	14306	8.58638E-06	0.001	19.791
	rs56221232	T	C	0.0828027	0.0167401	14306	7.61939E-07	0.002	24.467
	rs2017189	T	G	0.0506956	0.011024	14306	3.87258E-06	0.001	21.148
	rs62478070	T	G	0.224039	0.0494959	14306	5.93772E-06	0.001	20.488
	rs4962426	T	G	-0.0614216	0.0135979	14306	7.38482E-06	0.001	20.403
	rs7322368	C	T	-0.0815733	0.0183167	14306	5.51785E-06	0.001	19.834
	rs12585793	T	C	-0.262206	0.0564729	14306	5.79189E-06	0.002	21.558
	rs75238760	T	A	0.0619423	0.0139942	14306	6.79704E-06	0.001	19.592
unknown genus id.2071 (P<1×10^-5^)	rs4644504	T	C	-0.0969321	0.0216146	14306	5.81969E-06	0.001	20.111
	rs11809762	G	A	-0.0934634	0.0190198	14306	1.68287E-06	0.002	24.147
	rs11904514	A	G	0.109498	0.0249839	14306	7.89951E-06	0.001	19.208
	rs1809136	C	G	-0.0994594	0.0228638	14306	8.36989E-06	0.001	18.923
	rs16823675	C	T	-0.0767515	0.0149973	14306	2.33346E-07	0.002	26.191
	rs11684166	A	G	-0.0769635	0.0168349	14306	3.49116E-06	0.001	20.900
	rs10200320	T	C	-0.0641139	0.0142769	14306	5.6607E-06	0.001	20.167
	rs2898979	G	C	0.0901515	0.0202199	14306	7.67291E-06	0.001	19.879
	rs35740166	C	T	-0.112246	0.0226824	14306	8.39982E-07	0.002	24.489
	rs17086536	C	A	-0.100851	0.022432	14306	3.3638E-06	0.001	20.213
	rs34985298	G	A	-0.0623526	0.013772	14306	8.33758E-06	0.001	20.498
	rs1455639	A	G	-0.0760307	0.0169831	14306	7.83899E-06	0.001	20.042
	rs11195523	C	A	-0.0689278	0.0145351	14306	2.40121E-06	0.002	22.488
	rs2939766	A	G	-0.0591611	0.013042	14306	7.01148E-06	0.001	20.577
	rs76532867	T	C	0.112353	0.0242364	14306	2.55859E-06	0.001	21.490
	rs56975773	T	A	0.113282	0.0248859	14306	7.65491E-06	0.001	20.721
	rs12147596	C	T	-0.0719818	0.0141609	14306	2.86207E-07	0.002	25.838
	rs72700702	T	C	-0.091726	0.0189005	14306	1.59272E-06	0.002	23.553
	rs72707147	C	T	0.110109	0.0244644	14306	6.84022E-06	0.001	20.257
	rs6007642	C	T	-0.0791412	0.0177958	14306	9.95543E-06	0.001	19.777
family Clostridiales vadin BB60 group id.11286	rs7538034	T	G	-0.078598	0.0165982	14306	2.36706E-06	0.002	22.423
(P<1×10^-5^)	rs6588624	A	G	0.0662317	0.0138147	14306	1.79287E-06	0.002	22.985
	rs13409132	A	G	-0.165419	0.0352154	14306	4.3723E-06	0.002	22.065
	rs2191834	T	G	-0.0746375	0.0159136	14306	2.50196E-06	0.002	21.998
	rs6755871	C	G	-0.0613825	0.0138976	14306	9.33061E-06	0.001	19.508
	rs989682	A	G	0.070194	0.0155364	14306	6.84715E-06	0.001	20.413
	rs10517600	G	T	-0.0626993	0.0139364	14306	6.82763E-06	0.001	20.241
	rs34088226	A	G	-0.117807	0.026924	14306	7.66214E-06	0.001	19.145
	rs7725895	A	G	-0.116224	0.0240367	14306	3.94357E-06	0.002	23.380
	rs66714985	A	C	0.116908	0.0252447	14306	4.85333E-06	0.001	21.446
	rs118104867	C	T	0.214464	0.0455098	14306	3.43598E-06	0.002	22.207
	rs10904722	C	T	-0.0672314	0.0147123	14306	5.04836E-06	0.001	20.883
	rs17121075	G	A	0.0769254	0.0172234	14306	7.91425E-06	0.001	19.948
	rs55682560	C	T	-0.131519	0.0261319	14306	4.97038E-07	0.002	25.330
	rs28691777	C	T	0.137134	0.0266996	14306	6.95697E-07	0.002	26.380
	rs7226487	A	G	-0.0643682	0.0138701	14306	3.58286E-06	0.002	21.537
	rs9979874	G	C	-0.0738925	0.0150911	14306	1.05271E-06	0.002	23.975
unknown genus id.1000000073 (P<1×10^-5^)	rs6588624	A	G	0.0662317	0.0138147	14306	1.79287E-06	0.002	22.985
	rs7538034	T	G	-0.078598	0.0165982	14306	2.36706E-06	0.002	22.423
	rs2191834	T	G	-0.0746375	0.0159136	14306	2.50196E-06	0.002	21.998
	rs13409132	A	G	-0.165419	0.0352154	14306	4.3723E-06	0.002	22.065
	rs6755871	C	G	-0.0613825	0.0138976	14306	9.33061E-06	0.001	19.508
	rs989682	A	G	0.070194	0.0155364	14306	6.84715E-06	0.001	20.413
	rs10517600	G	T	-0.0626993	0.0139364	14306	6.82763E-06	0.001	20.241
	rs7725895	A	G	-0.116224	0.0240367	14306	3.94357E-06	0.002	23.380
	rs34088226	A	G	-0.117807	0.026924	14306	7.66214E-06	0.001	19.145
	rs66714985	A	C	0.116908	0.0252447	14306	4.85333E-06	0.001	21.446
	rs118104867	C	T	0.214464	0.0455098	14306	3.43598E-06	0.002	22.207
	rs10904722	C	T	-0.0672314	0.0147123	14306	5.04836E-06	0.001	20.883
	rs17121075	G	A	0.0769254	0.0172234	14306	7.91425E-06	0.001	19.948
	rs55682560	C	T	-0.131519	0.0261319	14306	4.97038E-07	0.002	25.330
	rs28691777	C	T	0.137134	0.0266996	14306	6.95697E-07	0.002	26.380
	rs7226487	A	G	-0.0643682	0.0138701	14306	3.58286E-06	0.002	21.537
	rs9979874	G	C	-0.0738925	0.0150911	14306	1.05271E-06	0.002	23.975
genus Clostridium innocuum group id.14397	rs6577484	G	A	0.160425	0.0360857	14306	8.40601E-06	0.001	19.764
(P<1×10^-5^)	rs1948423	T	A	-0.108859	0.023425	14306	3.49406E-06	0.002	21.596
	rs40656	C	T	0.142664	0.0311021	14306	8.61529E-06	0.001	21.040
	rs6890185	C	T	-0.113424	0.0233137	14306	1.12243E-06	0.002	23.669
	rs4869133	G	A	-0.180591	0.0409505	14306	7.24453E-06	0.001	19.448
	rs10074000	T	C	-0.102648	0.0227508	14306	6.99939E-06	0.001	20.357
	rs71564433	T	A	-0.126746	0.0274657	14306	7.8001E-06	0.001	21.295
	rs10506058	A	G	0.0997048	0.0221926	14306	8.92442E-06	0.001	20.184
	rs77845139	A	G	-0.114993	0.0257186	14306	8.40621E-06	0.001	19.992
	rs61267978	T	C	0.14708	0.0320875	14306	5.58509E-06	0.001	21.010
	rs1942371	G	A	-0.157938	0.034187	14306	4.0634E-06	0.001	21.343
class Lentisphaeria id.2250 (P<1×10^-5^)	rs72640280	A	G	0.220207	0.0486196	14306	5.18036E-06	0.001	20.513
	rs73113483	T	A	-0.131217	0.0288713	14306	8.66343E-06	0.001	20.656
	rs2731834	G	C	-0.109438	0.023693	14306	4.24356E-06	0.001	21.335
	rs11770843	C	T	0.109431	0.0234879	14306	1.9073E-06	0.002	21.707
	rs62570196	C	T	-0.21635	0.0439866	14306	1.07924E-06	0.002	24.192
	rs2031282	A	G	0.122368	0.0270329	14306	4.38258E-06	0.001	20.490
	rs17114848	G	A	0.152377	0.0324332	14306	4.05864E-06	0.002	22.073
	rs1002941	A	G	-0.105025	0.0233484	14306	8.14836E-06	0.001	20.234
	rs77599476	A	G	0.230292	0.0480168	14306	1.86132E-06	0.002	23.002
	rs2825714	A	G	-0.13741	0.0289246	14306	1.72211E-06	0.002	22.568
order Victivallales id.2254 (P<1×10^-5^)	rs72640280	A	G	0.220207	0.0486196	14306	5.18036E-06	0.001	20.513
	rs73113483	T	A	-0.131217	0.0288713	14306	8.66343E-06	0.001	20.656
	rs2731834	G	C	-0.109438	0.023693	14306	4.24356E-06	0.001	21.335
	rs11770843	C	T	0.109431	0.0234879	14306	1.9073E-06	0.002	21.707
	rs62570196	C	T	-0.21635	0.0439866	14306	1.07924E-06	0.002	24.192
	rs2031282	A	G	0.122368	0.0270329	14306	4.38258E-06	0.001	20.490
	rs1002941	A	G	-0.105025	0.0233484	14306	8.14836E-06	0.001	20.234
	rs17114848	G	A	0.152377	0.0324332	14306	4.05864E-06	0.002	22.073
	rs77599476	A	G	0.230292	0.0480168	14306	1.86132E-06	0.002	23.002
	rs2825714	A	G	-0.13741	0.0289246	14306	1.72211E-06	0.002	22.568
genus Bifidobacterium id.436 (P<1×10^-5^)	rs12022129	A	G	-0.0619356	0.0138937	14306	7.9965E-06	0.001	19.872
	rs1961273	C	T	0.0674036	0.0132319	14306	3.50865E-07	0.002	25.949
	rs13020688	G	A	0.0562696	0.0122617	14306	4.07258E-06	0.001	21.059
	rs182549	T	C	-0.119703	0.0127294	14306	1.2782E-20	0.006	88.429
	rs62181700	G	A	-0.0624643	0.0131205	14306	2.17245E-06	0.002	22.665
	rs4567981	T	A	0.0562084	0.0117923	14306	1.92832E-06	0.002	22.720
	rs55888705	A	G	0.0546319	0.0121139	14306	6.67022E-06	0.001	20.339
	rs4957061	T	C	0.0534239	0.0117431	14306	5.77936E-06	0.001	20.697
	rs73797465	T	G	-0.0953566	0.0209236	14306	4.38157E-06	0.001	20.770
	rs76671854	C	G	-0.0846055	0.0184003	14306	3.95667E-06	0.001	21.142
	rs857444	C	T	0.0558234	0.0121219	14306	0.000003571	0.001	21.208
	rs2686790	C	T	-0.070741	0.0157926	14306	7.49894E-06	0.001	20.065
	rs2491158	A	G	-0.0712624	0.015983	14306	8.04711E-06	0.001	19.879
	rs10841473	G	C	-0.0624207	0.0129438	14306	1.6452E-06	0.002	23.256
	rs7322849	T	C	0.112428	0.0201813	14306	1.08368E-08	0.002	31.035
	rs540489	T	G	-0.0637641	0.0138746	14306	5.19457E-06	0.001	21.121
	rs75344046	C	T	0.232354	0.0505979	14306	4.86351E-06	0.001	21.088
	rs5746486	T	C	-0.0536216	0.0120801	14306	8.99953E-06	0.001	19.703

SNPs, single nucleotide polymorphisms; EA, effect allele; OA, other allele; Beta, effect estimate; SE, standard error
