# Supplementary material for: Altered Gut Microbiota as a Potential Risk Factor for Coronary Artery Disease in Diabetes: A Two-Sample Bi-Directional Mendelian Randomization Study
Source: Int J Med Sci. 2024 Jan 1;21(2):376–95. doi: 10.7150/ijms.92131 (PMC10758148; doi:10.7150/ijms.92131)
Supplement: Supplementary file 1 — Supplementary figures and tables. [file ijmsv21p0376s1.pdf]

**Supplementary Materials for**

**This file includes:**

**Table S1.** Description of Type 2 diabetes, Coronary artery disease, gut microbiota and metabolite.

**Table S2.** Results of a bidirectional Mendelian randomization analysis of type 2 diabetes ($P < 5\times10^{-8}$) and coronary artery disease ($P < 5\times10^{-8}$).

**Table S3.** MR Results of metabolites on T2D.

**Table S4.** MR Results of metabolites on CAD.

**Table S5.** Particulars of SNPs used in MR analyses of gut microbiota.

**Table S6.** MR Power calculation for detecting significant ($P < 0.05$) causal effect of gut microbiota on the risk of T2D/CAD, and T2D/CAD on the risk of gut microbiota.

**Figure S1.** Leave-one-out analysis of inverse-variance weighted (IVW) estimates between genetically determined gut microbiota traits ($p < 1 \times 10^{-5}$) and Type 2 diabetes (T2D), as well as coronary artery disease (CAD).

**Figure S2.** Leave-one-out analysis of inverse-variance weighted (IVW) estimates between genetically determined type 2 diabetes (T2D) traits ($P < 5\times10^{-8}$), as well as coronary artery disease (CAD) traits ($P < 5\times10^{-8}$), and gut microbiota.

**Figure S3.** Leave-one-out analysis of inverse-variance weighted (IVW) estimates between genetically determined metabolites traits ($p < 1 \times 10^{-5}$) and Type 2 diabetes (T2D), as well as coronary artery disease (CAD).

**Additional information 1.** Harmonized data**.**

**Additional information 2.** Results of MR analysis.

**Table S1. Description of Type 2 diabetes, Coronary artery disease, gut microbiota and metabolite.**

| Traits | Consortium | First author | Sample size | Populations | Journal | Year | Download link |
|---|---|---|---|---|---|---|---|
| Type 2 diabetes | NA | Xue A | 655,666 (61,714 cases / 1,178 controls) | European ancestry | Nat Commun. | 2018 | https://gwas.mrcieu.ac.uk/datasets/ebi-a-GCST006867/ |
| Coronary artery disease | NA | Nikpay M | 141,217 (42,096 cases / 361 controls) | European ancestry | Nat Genet. | 2015 | https://gwas.mrcieu.ac.uk/datasets/ebi-a-GCST003116/ |
| Gut microbiota | MiBioGen[a] | Kurilshikov | 18,340 | predominantly European ancestry | Nat Genet. | 2020 | https://mibiogen.gcc.rug.nl/ |
| Metabolites | FHS | Rhee | 2076 | European ancestry | Cell Metab. | 2013 | https://www.cell.com/cell-metabolism/fulltext/S1550-4131(13)00257-X?_returnURL=https%3A%2F%2Flinkinghub.elsevier.com%2Fretrieve%2Fpii%2FS155041311300257X%3Fshowall%3Dtrue#secsectitle0110 |

[a] MiBioGen consortium curated and analyzed genome-wide genotypes and 16S fecal microbiome data from 18,340 individuals (24 cohorts). Twenty cohorts included samples of single ancestry, namely European (16 cohorts; n= 13,266), Middle Eastern (1 cohort; n= 481), East Asian (1 cohort; n= 811), American Hispanic/Latin (1 cohort; n= 1,097) and African American (1 cohort; n= 114), whereas four cohorts included samples from multiple ancestries (n = 2,571).

**Table S2. Results of a bidirectional Mendelian randomization analysis of type 2 diabetes (P<5×10$^{-8}$) and coronary artery disease (P<5×10$^{-8}$).**

| Exposure | Outcome | Method | NSNP | Beta | P | Directional pleiotropy | | Cochrane's Q-statistic(P) | Steiger P | IVW MRE |
|---|---|---|---|---|---|---|---|---|---|---|
| | | | | | | Egger intercept(P) | MRPRESSO RSSobs(P) | | | |
| CAD | T2D | MR Egger | 29 | -0.005 (-0.219,0.210) | 9.67E-01 | 0.007 (0.495) | 148.952 (<0.001) | 131.446 (2.44E-15) | 6.17E-152 | 0.104 |
| | | Weighted median | 29 | 0.062 (-0.011,0.135) | 9.67E-02 | | | | | |
| | | IVW | 29 | 0.066 (-0.014,0.145) | 1.04E-01 | | | | | |
| T2D | CAD | MR Egger | 134 | 0.074 (-0.015,0.163) | 1.07E-01 | 0.006 (0.058) | 308.837 (<0.001) | 303.207 (2.59E-15) | 0 | 3.51E-14 |
| | | Weighted median | 134 | 0.110 (0.057,0.163) | 4.50E-05 | | | | | |
| | | IVW | 134 | 0.152 (0.113,0.191) | 3.51E-14 | | | | | |

T2D, Type 2 diabetes; CAD, Coronary artery disease; IVW, inverse variance weighted; NSNPs, number of single nucleotide polymorphisms; beta, mendelian randomization effect estimate; se, standard error of mendelian randomization effect estimate; OR(95%-CI), odds ratio and 95% confidence interval of odds ratio estimate; IVW MRE, Inverse variance weighted (multiplicative random effects)

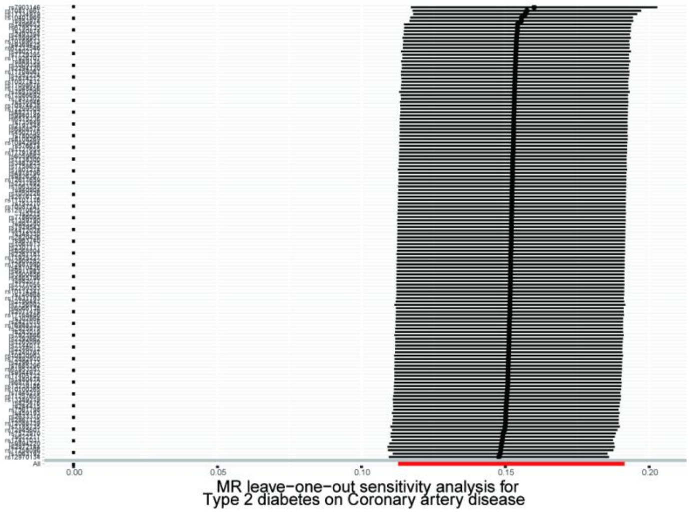


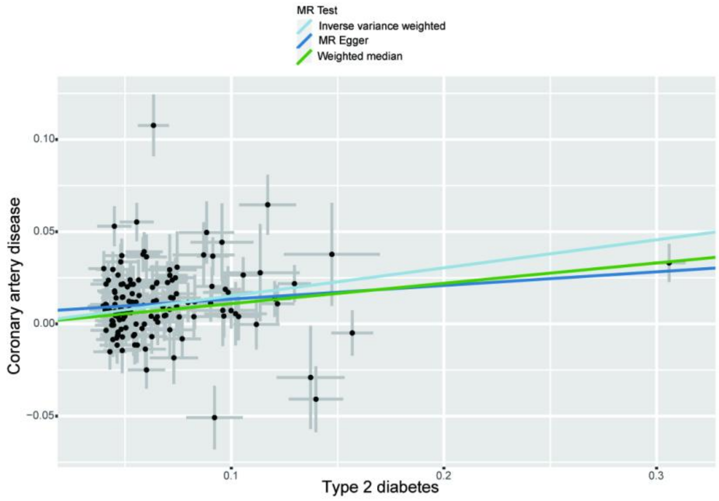


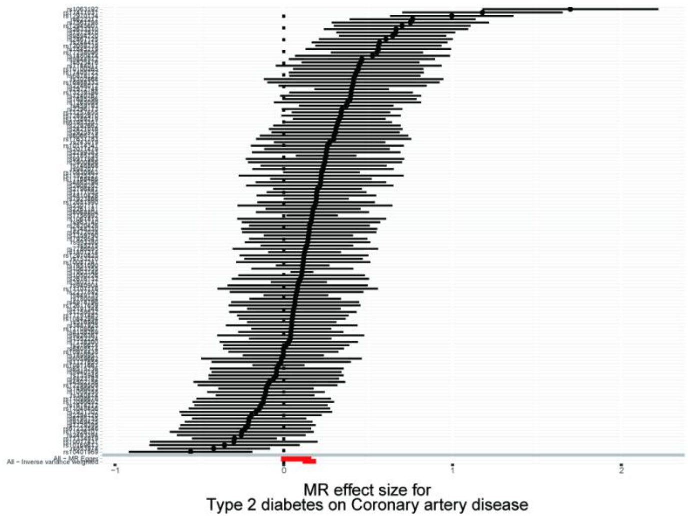


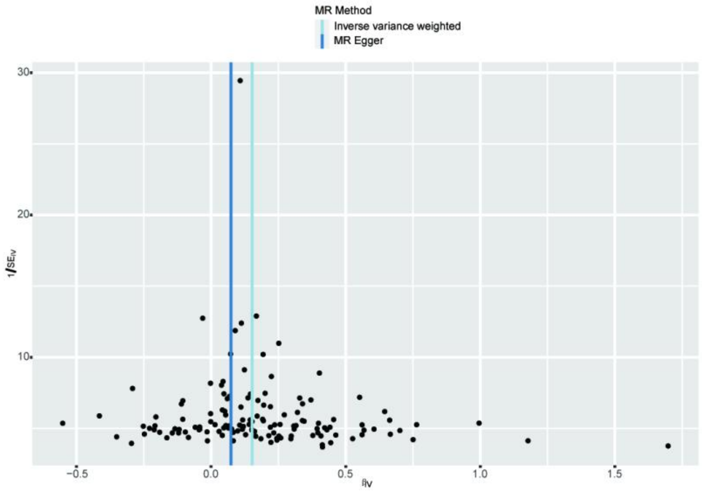

**Table S3. MR Results of metabolites on T2D.**

| Exposure | Outcome | Method | NSNP | Beta (95%CI) | P | Directional pleiotropy | | Cochrane's Q-statistic(P) | Steiger P |
|---|---|---|---|---|---|---|---|---|---|
| | | | | | | Egger intercept(P) | MRPRESSO RSSobs(P) | | |
| SM 14_0 | T2D | MR Egger | 68 | -0.006(-0.041,0.029) | 0.735 | -0.007(0.152) | 73.772(0.37) | 71.865(0.32) | 0 |
| | | Weighted median | 68 | -0.03(-0.044,-0.016) | <0.001 | | | | |
| | | IVW | 68 | -0.031(-0.041,-0.02) | <0.001 | | | | |
| salicylurate | T2D | MR Egger | 56 | -0.022(-0.056,0.011) | 0.197 | 0(0.925) | 49.032(0.78) | 47.374(0.758) | 0 |
| | | Weighted median | 56 | -0.021(-0.038,-0.005) | 0.012 | | | | |
| | | IVW | 56 | -0.024(-0.035,-0.013) | <0.001 | | | | |
| proline | T2D | MR Egger | 24 | -0.067(-0.139,0.006) | 0.084 | 0.012(0.364) | 29.089(0.276) | 27.085(0.252) | 8.21E-216 |
| | | Weighted median | 24 | -0.051(-0.072,-0.031) | <0.001 | | | | |
| | | IVW | 24 | -0.034(-0.05,-0.017) | <0.001 | | | | |
| LPC 18_2 | T2D | MR Egger | 41 | -0.1(-0.189,-0.011) | 0.034 | 0.014(0.153) | 56.839(0.074) | 54.044(0.068) | 1.60E-228 |
| | | Weighted median | 41 | -0.059(-0.082,-0.037) | <0.001 | | | | |
| | | IVW | 41 | -0.035(-0.052,-0.018) | <0.001 | | | | |
| citrulline | T2D | MR Egger | 14 | -0.143(-0.213,-0.072) | 0.002 | 0.036(0.024) | 18.286(0.327) | 15.727(0.264) | 2.19E-67 |
| | | Weighted median | 14 | -0.072(-0.112,-0.033) | <0.001 | | | | |
| | | IVW | 14 | -0.057(-0.087,-0.027) | <0.001 | | | | |
| glycerol | T2D | MR Egger | 38 | -0.022(-0.065,0.022) | 0.343 | -0.001(0.882) | 24.241(0.976) | 22.807(0.968) | 1.03E-255 |
| | | Weighted median | 38 | -0.022(-0.042,-0.003) | 0.022 | | | | |
| | | IVW | 38 | -0.025(-0.039,-0.011) | <0.001 | | | | |
| ornithine | T2D | MR Egger | 72 | -0.048(-0.091,-0.005) | 0.031 | 0.007(0.186) | 63.115(0.823) | 61.124(0.792) | 0 |
| | | Weighted median | 72 | -0.025(-0.042,-0.009) | 0.002 | | | | |
| | | IVW | 72 | -0.02(-0.031,-0.009) | 0.001 | | | | |
| taurocholate | T2D | MR Egger | 58 | 0.049(0.019,0.078) | 0.002 | -0.017(0.022) | 33.341(0.996) | 31.997(0.997) | 0 |
| | | Weighted median | 58 | 0.025(0.012,0.038) | <0.001 | | | | |
| | | IVW | 58 | 0.015(0.006,0.025) | 0.002 | | | | |
| adenosine monophosphate | T2D | MR Egger | 24 | -0.034(-0.095,0.026) | 0.280 | 0.001(0.84) | 11.649(0.996) | 9.267(0.995) | 5.99E-139 |
| | | Weighted median | 24 | -0.039(-0.062,-0.016) | 0.001 | | | | |
| | | IVW | 24 | -0.028(-0.046,-0.01) | 0.002 | | | | |

| | | | | | | | | | |
|---|---|---|---|---|---|---|---|---|---|
| cystathionine | T2D | MR Egger | 21 | -0.131(-0.209,-0.053) | 0.004 | 0.028(0.017) | 20.203(0.605) | 18.002(0.587) | 2.42E-98 |
| | | Weighted median | 21 | -0.045(-0.073,-0.017) | 0.001 | | | | |
| | | IVW | 21 | -0.03(-0.049,-0.011) | 0.002 | | | | |
| serotonin | T2D | MR Egger | 25 | -0.192(-0.461,0.077) | 0.174 | 0.033(0.258) | 38.98(0.062) | 35.716(0.058) | 1.41E-117 |
| | | Weighted median | 25 | -0.015(-0.044,0.014) | 0.314 | | | | |
| | | IVW | 25 | -0.034(-0.057,-0.01) | 0.005 | | | | |
| asparagine | T2D | MR Egger | 46 | -0.006(-0.056,0.045) | 0.829 | -0.003(0.654) | 42.776(0.708) | 34.805(0.864) | 0 |
| | | Weighted median | 46 | -0.016(-0.034,0.003) | 0.094 | | | | |
| | | IVW | 46 | -0.017(-0.03,-0.004) | 0.013 | | | | |
| adenosine | T2D | MR Egger | 8 | -0.162(-0.359,0.036) | 0.159 | 0.025(0.266) | 4.843(0.826) | 3.708(0.813) | 3.04E-34 |
| | | Weighted median | 8 | -0.043(-0.085,0) | 0.048 | | | | |
| | | IVW | 8 | -0.04(-0.074,-0.007) | 0.019 | | | | |
| TAG 58_8 | T2D | MR Egger | 37 | 0.053(-0.039,0.146) | 0.264 | -0.015(0.123) | 44.58(0.239) | 42.146(0.222) | 5.71E-241 |
| | | Weighted median | 37 | -0.029(-0.051,-0.006) | 0.012 | | | | |
| | | IVW | 37 | -0.02(-0.036,-0.003) | 0.019 | | | | |
| PC 36_1 | T2D | MR Egger | 22 | 0.041(-0.18,0.262) | 0.719 | -0.003(0.868) | 21.319(0.671) | 16.577(0.736) | 2.33E-145 |
| | | Weighted median | 22 | 0.014(-0.01,0.039) | 0.252 | | | | |
| | | IVW | 22 | 0.022(0.003,0.041) | 0.022 | | | | |
| suberic acid | T2D | MR Egger | 4 | 0.109(-0.455,0.672) | 0.742 | -0.018(0.848) | 1.096(0.902) | 0.465(0.927) | 4.80E-18 |
| | | Weighted median | 4 | 0.04(-0.009,0.089) | 0.113 | | | | |
| | | IVW | 4 | 0.046(0.006,0.086) | 0.023 | | | | |

MR, mendelian randomization; T2D, Type 2 diabetes; IVW, inverse variance weighted; NSNPs, number of single nucleotide polymorphisms; beta, mendelian randomization effect estimate; CI, confidence interval; RSSobs, residual sums of squares of observations; SM, sphingomyelin; LPC lysophosphatidylcholine; TAG, triacylglycerol; PC, phosphatidylcholine.

**Table S4. MR Results of metabolites on CAD.**

| Exposure | Outcome | Method | NSNP | Beta (95%CI) | P | Directional pleiotropy | | Cochrane's Q-statistic(P) | Steiger P |
|---|---|---|---|---|---|---|---|---|---|
| | | | | | | Egger intercept(P) | MRPRESSO RSSobs(P) | | |
| 3_hydroxyphenylacetic acid | CAD | MR Egger | 18 | -0.095(-0.206,0.016) | 0.114 | 0.025(0.047) | 19.849(0.477) | 17.117(0.446) | 1.82E-82 |
| | | Weighted median | 18 | 0.021(-0.015,0.056) | 0.252 | | | | |
| | | IVW | 18 | 0.024(-0.001,0.049) | 0.057 | | | | |
| lysine | CAD | MR Egger | 12 | -0.083(-0.182,0.015) | 0.129 | 0.009(0.524) | 7.878(0.851) | 6.756(0.818) | 1.17E-51 |
| | | Weighted median | 12 | -0.063(-0.097,-0.028) | <0.001 | | | | |
| | | IVW | 12 | -0.051(-0.078,-0.025) | <0.001 | | | | |
| alpha_ketoglutarate | CAD | MR Egger | 42 | -0.003(-0.047,0.04) | 0.878 | -0.007(0.248) | 33.428(0.873) | 31.813(0.848) | 1.41E-249 |
| | | Weighted median | 42 | -0.042(-0.064,-0.02) | <0.001 | | | | |
| | | IVW | 42 | -0.027(-0.043,-0.011) | 0.001 | | | | |
| LPC 18_2 | CAD | MR Egger | 44 | 0.085(0.01,0.16) | 0.032 | -0.013(0.138) | 48.301(0.354) | 46.235(0.34) | 1.02E-248 |
| | | Weighted median | 44 | 0.016(-0.009,0.041) | 0.218 | | | | |
| | | IVW | 44 | 0.029(0.011,0.047) | 0.002 | | | | |
| CE 18_2 | CAD | MR Egger | 15 | 0.035(-0.058,0.127) | 0.476 | 0.002(0.869) | 9.655(0.89) | 8.478(0.863) | 6.46E-70 |
| | | Weighted median | 15 | 0.049(0.012,0.087) | 0.010 | | | | |
| | | IVW | 15 | 0.042(0.014,0.07) | 0.003 | | | | |
| guanosine diphosphate | CAD | MR Egger | 47 | -0.019(-0.091,0.052) | 0.595 | 0(0.982) | 32.725(0.964) | 31.292(0.952) | 0 |
| | | Weighted median | 47 | -0.036(-0.055,-0.017) | <0.001 | | | | |
| | | IVW | 47 | -0.02(-0.034,-0.006) | 0.004 | | | | |
| proline | CAD | MR Egger | 39 | -0.008(-0.059,0.043) | 0.760 | -0.004(0.604) | 39.739(0.476) | 37.746(0.481) | 0 |
| | | Weighted median | 39 | -0.023(-0.044,-0.002) | 0.031 | | | | |
| | | IVW | 39 | -0.021(-0.036,-0.007) | 0.004 | | | | |
| carnitine | CAD | MR Egger | 71 | -0.064(-0.096,-0.033) | <0.001 | 0.015(0.001) | 56.262(0.927) | 54.978(0.906) | NA |
| | | Weighted median | 71 | -0.016(-0.03,-0.003) | 0.019 | | | | |
| | | IVW | 71 | -0.014(-0.024,-0.004) | 0.005 | | | | |

| | | | | | | | | | |
|---|---|---|---|---|---|---|---|---|---|
| hypoxanthine | CAD | MR Egger | 21 | 0.064(0.001,0.126) | 0.061 | -0.008(0.263) | 9.564(0.988) | 8.698(0.986) | 4.43E-102 |
| | | Weighted median | 21 | 0.037(0.009,0.065) | 0.009 | | | | |
| | | IVW | 21 | 0.029(0.008,0.05) | 0.007 | | | | |
| salicylurate | CAD | MR Egger | 59 | 0.001(-0.016,0.018) | 0.942 | 0.005(0.087) | 36.548(0.992) | 34.661(0.994) | 0 |
| | | Weighted median | 59 | 0.005(-0.011,0.022) | 0.533 | | | | |
| | | IVW | 59 | 0.013(0.003,0.023) | 0.008 | | | | |
| aspartate | CAD | MR Egger | 17 | 0.034(-0.008,0.075) | 0.130 | -0.003(0.621) | 8.855(0.973) | 6.47(0.982) | 5.29E-89 |
| | | Weighted median | 17 | 0.031(0.006,0.055) | 0.015 | | | | |
| | | IVW | 17 | 0.024(0.006,0.043) | 0.010 | | | | |
| anthranilic acid | CAD | MR Egger | 46 | 0.038(-0.005,0.081) | 0.092 | -0.004(0.409) | 45.065(0.599) | 42.363(0.584) | 2.86E-289 |
| | | Weighted median | 46 | 0.01(-0.013,0.032) | 0.403 | | | | |
| | | IVW | 46 | 0.021(0.005,0.037) | 0.011 | | | | |
| UDP glucose; UDP galactose | CAD | MR Egger | 50 | -0.025(-0.105,0.055) | 0.544 | 0.002(0.854) | 26.901(0.999) | 25.866(0.997) | 0 |
| | | Weighted median | 50 | -0.021(-0.04,-0.002) | 0.033 | | | | |
| | | IVW | 50 | -0.018(-0.032,-0.003) | 0.015 | | | | |
| allantoin | CAD | MR Egger | 32 | -0.054(-0.1,-0.008) | 0.028 | 0.012(0.126) | 18.482(0.977) | 17.4(0.976) | 1.22E-185 |
| | | Weighted median | 32 | -0.031(-0.053,-0.009) | 0.006 | | | | |
| | | IVW | 32 | -0.019(-0.035,-0.003) | 0.017 | | | | |
| quinolinic acid | CAD | MR Egger | 72 | -0.043(-0.074,-0.012) | 0.009 | 0.008(0.048) | 53.779(0.956) | 51.442(0.961) | 0 |
| | | Weighted median | 72 | -0.026(-0.041,-0.01) | 0.001 | | | | |
| | | IVW | 72 | -0.013(-0.024,-0.002) | 0.020 | | | | |
| asparagine | CAD | MR Egger | 49 | 0.047(-0.003,0.098) | 0.071 | -0.008(0.228) | 20.766(1) | 19.63(1) | 0 |
| | | Weighted median | 49 | 0.009(-0.011,0.03) | 0.362 | | | | |
| | | IVW | 49 | 0.017(0.003,0.032) | 0.021 | | | | |

MR, mendelian randomization; CAD, coronary artery disease ; IVW, inverse variance weighted; NSNPs, number of single nucleotide polymorphisms; beta, mendelian randomization effect estimate; CI, confidence interval; RSSobs, residual sums of squares of observations; LPC lysophosphatidylcholine; CE, cholesterol ester.

**Table 3. Particulars of SNPs used in MR analyses of gut microbiota.**

| Exposure traits | SNPs | EA | OA | Beta | Se | samplesize | P | $R^2$ | F-statistic |
|---|---|---|---|---|---|---|---|---|---|
| Type 2 diabetes ($P<5\times10^{-8}$) | rs1127655 | T | C | -0.0438 | 0.0079 | 62892 | 2.47E-08 | 0.000 | 30.739 |
| | rs12088739 | G | A | -0.0884 | 0.013 | 62892 | 9.79E-12 | 0.001 | 46.240 |
| | rs2493394 | G | A | 0.073 | 0.0113 | 62892 | 1.15E-10 | 0.001 | 41.734 |
| | rs348330 | A | G | -0.0487 | 0.0081 | 62892 | 1.86E-09 | 0.001 | 36.148 |
| | rs2820426 | G | A | 0.0521 | 0.0073 | 62892 | 1.30E-12 | 0.001 | 50.937 |
| | rs2296173 | G | A | 0.065 | 0.0087 | 62892 | 7.66E-14 | 0.001 | 55.820 |
| | rs340874 | C | T | 0.0626 | 0.0073 | 62892 | 8.41E-18 | 0.001 | 73.536 |
| | rs7572970 | G | A | 0.059 | 0.0087 | 62892 | 1.39E-11 | 0.001 | 45.990 |
| | rs1009358 | C | T | -0.0545 | 0.008 | 62892 | 9.81E-12 | 0.001 | 46.410 |
| | rs2972144 | G | A | 0.0913 | 0.0075 | 62892 | 2.55E-34 | 0.002 | 148.190 |
| | rs243019 | C | T | 0.0566 | 0.0071 | 62892 | 2.29E-15 | 0.001 | 63.550 |
| | rs780094 | C | T | 0.0692 | 0.0074 | 62892 | 5.16E-21 | 0.001 | 87.448 |
| | rs10169613 | T | C | -0.0429 | 0.0078 | 62892 | 3.57E-08 | 0.000 | 30.250 |
| | rs12617659 | T | C | -0.0685 | 0.0103 | 62892 | 2.83E-11 | 0.001 | 44.229 |
| | rs17334919 | T | C | -0.1398 | 0.0128 | 62892 | 6.69E-28 | 0.002 | 119.287 |
| | rs2867125 | C | T | 0.0601 | 0.0096 | 62892 | 4.33E-10 | 0.001 | 39.193 |
| | rs13389219 | T | C | -0.0722 | 0.0074 | 62892 | 2.11E-22 | 0.002 | 95.194 |
| | rs7561798 | G | A | 0.04 | 0.0072 | 62892 | 2.79E-08 | 0.000 | 30.864 |
| | rs3887925 | T | C | 0.0474 | 0.0079 | 62892 | 2.47E-09 | 0.001 | 36.000 |
| | rs9844972 | C | G | 0.0956 | 0.0148 | 62892 | 1.03E-10 | 0.001 | 41.725 |
| | rs6808574 | C | T | 0.0552 | 0.0076 | 62892 | 4.39E-13 | 0.001 | 52.753 |
| | rs11708067 | G | A | -0.0965 | 0.0086 | 62892 | 5.93E-29 | 0.002 | 125.909 |
| | rs9826367 | G | A | -0.0439 | 0.0079 | 62892 | 2.77E-08 | 0.000 | 30.880 |
| | rs2292662 | T | C | -0.0629 | 0.0111 | 62892 | 1.24E-08 | 0.001 | 32.111 |
| | rs6795735 | T | C | -0.0558 | 0.0073 | 62892 | 1.63E-14 | 0.001 | 58.428 |
| | rs7651090 | G | A | 0.1204 | 0.0076 | 62892 | 3.85E-57 | 0.004 | 250.972 |
| | rs11926707 | C | T | 0.0463 | 0.0082 | 62892 | 1.69E-08 | 0.001 | 31.881 |
| | rs1899951 | T | C | -0.1118 | 0.0109 | 62892 | 1.64E-24 | 0.002 | 105.204 |
| | rs11925227 | A | G | -0.0534 | 0.0095 | 62892 | 2.25E-08 | 0.001 | 31.596 |
| | rs4472028 | C | T | -0.0453 | 0.0071 | 62892 | 2.08E-10 | 0.001 | 40.708 |

| | | | | | | | | |
|---|---|---|---|---|---|---|---|---|
| rs1496653 | G | A | -0.0769 | 0.0088 | 62892 | 2.57E-18 | 0.001 | 76.364 |
| rs7674212 | T | G | -0.0465 | 0.0075 | 62892 | 6.18E-10 | 0.001 | 38.440 |
| rs993380 | G | A | -0.0507 | 0.0081 | 62892 | 4.59E-10 | 0.001 | 39.178 |
| rs11098676 | C | T | 0.054 | 0.0096 | 62892 | 2.03E-08 | 0.001 | 31.641 |
| rs1801214 | T | C | 0.0903 | 0.0074 | 62892 | 5.52E-34 | 0.002 | 148.906 |
| rs17086692 | T | G | -0.0467 | 0.0084 | 62892 | 2.48E-08 | 0.000 | 30.908 |
| rs7685296 | T | C | -0.0511 | 0.0081 | 62892 | 2.32E-10 | 0.001 | 39.799 |
| rs735949 | C | T | -0.0711 | 0.0106 | 62892 | 1.95E-11 | 0.001 | 44.991 |
| rs459193 | G | A | 0.0711 | 0.0083 | 62892 | 8.81E-18 | 0.001 | 73.381 |
| rs3900856 | A | G | 0.1136 | 0.0185 | 62892 | 7.35E-10 | 0.001 | 37.706 |
| rs6878122 | A | G | -0.0564 | 0.0079 | 62892 | 1.19E-12 | 0.001 | 50.969 |
| rs7729395 | T | C | 0.1373 | 0.016 | 62892 | 1.10E-17 | 0.001 | 73.638 |
| rs1061813 | A | G | -0.0429 | 0.0073 | 62892 | 3.37E-09 | 0.001 | 34.536 |
| rs4865796 | A | G | 0.053 | 0.0078 | 62892 | 1.33E-11 | 0.001 | 46.170 |
| rs2307111 | C | T | -0.0407 | 0.0074 | 62892 | 3.03E-08 | 0.000 | 30.250 |
| rs10077431 | A | C | -0.0487 | 0.0089 | 62892 | 4.75E-08 | 0.000 | 29.942 |
| rs853974 | C | T | -0.0601 | 0.0088 | 62892 | 7.86E-12 | 0.001 | 46.643 |
| rs746868 | G | C | 0.0519 | 0.0081 | 62892 | 1.40E-10 | 0.001 | 41.055 |
| rs9369425 | A | G | -0.0546 | 0.0085 | 62892 | 1.13E-10 | 0.001 | 41.262 |
| rs72892910 | T | G | 0.0648 | 0.0099 | 62892 | 6.43E-11 | 0.001 | 42.843 |
| rs7756992 | G | A | 0.1297 | 0.0078 | 62892 | 6.00E-62 | 0.004 | 276.497 |
| rs622217 | C | T | -0.0485 | 0.0077 | 62892 | 3.13E-10 | 0.001 | 39.674 |
| rs1063355 | G | T | 0.0709 | 0.0079 | 62892 | 3.72E-19 | 0.001 | 80.545 |
| rs1265099 | G | A | 0.0452 | 0.0074 | 62892 | 9.95E-10 | 0.001 | 37.309 |
| rs2071479 | T | C | 0.1473 | 0.0226 | 62892 | 6.62E-11 | 0.001 | 42.480 |
| rs2246012 | C | T | 0.0527 | 0.0094 | 62892 | 2.43E-08 | 0.000 | 31.432 |
| rs1050226 | G | A | -0.0491 | 0.0074 | 62892 | 3.34E-11 | 0.001 | 44.025 |
| rs17168486 | T | C | 0.0742 | 0.0094 | 62892 | 2.18E-15 | 0.001 | 62.309 |
| rs2908282 | A | G | 0.0552 | 0.0094 | 62892 | 4.25E-09 | 0.001 | 34.484 |
| rs2191348 | T | G | 0.0652 | 0.0073 | 62892 | 3.44E-19 | 0.001 | 79.772 |
| rs7786095 | G | A | -0.0743 | 0.0129 | 62892 | 9.64E-09 | 0.001 | 33.174 |
| rs13239186 | T | C | 0.0539 | 0.0085 | 62892 | 2.70E-10 | 0.001 | 40.211 |

| | | | | | | | | |
|---|---|---|---|---|---|---|---|---|
| rs13234269 | A | T | -0.0583 | 0.0078 | 62892 | 6.98E-14 | 0.001 | 55.866 |
| rs2299383 | T | C | 0.0412 | 0.0073 | 62892 | 1.49E-08 | 0.001 | 31.853 |
| rs849135 | A | G | -0.0999 | 0.0072 | 62892 | 1.04E-43 | 0.003 | 192.516 |
| rs10100265 | C | A | -0.0491 | 0.0079 | 62892 | 6.29E-10 | 0.001 | 38.629 |
| rs2294120 | G | A | -0.0443 | 0.0079 | 62892 | 1.62E-08 | 0.000 | 31.445 |
| rs7845219 | C | T | -0.0422 | 0.0072 | 62892 | 4.54E-09 | 0.001 | 34.353 |
| rs12681990 | C | T | 0.0634 | 0.0096 | 62892 | 3.62E-11 | 0.001 | 43.615 |
| rs10087241 | A | G | -0.0475 | 0.008 | 62892 | 2.80E-09 | 0.001 | 35.254 |
| rs3802177 | A | G | -0.1217 | 0.008 | 62892 | 2.32E-52 | 0.004 | 231.420 |
| rs516946 | C | T | 0.0824 | 0.0085 | 62892 | 3.16E-22 | 0.001 | 93.976 |
| rs17411031 | G | C | -0.045 | 0.0081 | 62892 | 3.04E-08 | 0.000 | 30.864 |
| rs10974438 | C | A | 0.0591 | 0.0075 | 62892 | 3.01E-15 | 0.001 | 62.094 |
| rs17791483 | G | A | -0.102 | 0.0147 | 62892 | 3.42E-12 | 0.001 | 48.147 |
| rs10811661 | C | T | -0.1569 | 0.0098 | 62892 | 4.13E-58 | 0.004 | 256.327 |
| rs2796441 | A | G | -0.0715 | 0.0073 | 62892 | 1.96E-22 | 0.002 | 95.933 |
| rs1758632 | G | C | 0.0491 | 0.0081 | 62892 | 1.36E-09 | 0.001 | 36.745 |
| rs10114341 | C | T | -0.0409 | 0.0072 | 62892 | 1.15E-08 | 0.001 | 32.269 |
| rs1063192 | A | G | 0.0634 | 0.0073 | 62892 | 3.30E-18 | 0.001 | 75.428 |
| rs2633310 | T | G | -0.0443 | 0.0079 | 62892 | 2.37E-08 | 0.000 | 31.445 |
| rs4918796 | C | T | 0.0623 | 0.0086 | 62892 | 4.01E-13 | 0.001 | 52.478 |
| rs7923866 | T | C | -0.0972 | 0.0074 | 62892 | 9.34E-40 | 0.003 | 172.532 |
| rs2421016 | T | C | -0.0458 | 0.0071 | 62892 | 1.48E-10 | 0.001 | 41.612 |
| rs753270 | C | T | 0.0528 | 0.0079 | 62892 | 2.70E-11 | 0.001 | 44.670 |
| rs11257655 | T | C | 0.0737 | 0.0087 | 62892 | 1.97E-17 | 0.001 | 71.762 |
| rs7903146 | T | C | 0.3059 | 0.0077 | 62892 | 1.00E-200 | 0.024 | 1578.256 |
| rs11591741 | C | G | -0.0475 | 0.0078 | 62892 | 1.23E-09 | 0.001 | 37.085 |
| rs2616132 | A | G | 0.0455 | 0.0078 | 62892 | 6.58E-09 | 0.001 | 34.028 |
| rs7929543 | C | A | 0.0828 | 0.0138 | 62892 | 2.20E-09 | 0.001 | 36.000 |
| rs7931302 | A | C | -0.0461 | 0.008 | 62892 | 7.65E-09 | 0.001 | 33.206 |
| rs67232546 | T | C | 0.0596 | 0.0096 | 62892 | 4.66E-10 | 0.001 | 38.543 |
| rs10830963 | G | C | 0.0909 | 0.008 | 62892 | 5.85E-30 | 0.002 | 129.106 |
| rs1552224 | C | A | -0.1034 | 0.0101 | 62892 | 8.64E-25 | 0.002 | 104.809 |

| | | | | | | | | |
|---|---|---|---|---|---|---|---|---|
| rs2237892 | T | C | -0.096 | 0.0157 | 62892 | 8.75E-10 | 0.001 | 37.389 |
| rs5215 | T | C | -0.0678 | 0.0073 | 62892 | 2.09E-20 | 0.001 | 86.261 |
| rs10842994 | T | C | -0.0755 | 0.0091 | 62892 | 1.02E-16 | 0.001 | 68.835 |
| rs2261181 | T | C | 0.0985 | 0.0118 | 62892 | 9.18E-17 | 0.001 | 69.680 |
| rs11107116 | T | G | 0.0467 | 0.0085 | 62892 | 3.75E-08 | 0.000 | 30.185 |
| rs11048456 | T | C | -0.0488 | 0.0082 | 62892 | 2.97E-09 | 0.001 | 35.417 |
| rs940904 | A | G | 0.0499 | 0.0083 | 62892 | 2.08E-09 | 0.001 | 36.145 |
| rs1480474 | G | A | 0.0411 | 0.0073 | 62892 | 1.68E-08 | 0.001 | 31.698 |
| rs12299509 | G | A | 0.0467 | 0.0073 | 62892 | 2.09E-10 | 0.001 | 40.925 |
| rs825476 | T | C | 0.0524 | 0.0073 | 62892 | 6.80E-13 | 0.001 | 51.525 |
| rs7138300 | T | C | -0.0443 | 0.0072 | 62892 | 5.65E-10 | 0.001 | 37.857 |
| rs61953351 | T | G | -0.07 | 0.0091 | 62892 | 1.98E-14 | 0.001 | 59.172 |
| rs963740 | T | A | -0.0479 | 0.0086 | 62892 | 2.23E-08 | 0.000 | 31.022 |
| rs576674 | A | G | -0.0654 | 0.0097 | 62892 | 1.79E-11 | 0.001 | 45.458 |
| rs1359790 | A | G | -0.0796 | 0.008 | 62892 | 2.80E-23 | 0.002 | 99.003 |
| rs7144011 | T | G | 0.0482 | 0.0085 | 62892 | 1.64E-08 | 0.001 | 32.156 |
| rs12910825 | G | A | 0.0517 | 0.0074 | 62892 | 2.16E-12 | 0.001 | 48.811 |
| rs982077 | G | A | -0.0453 | 0.0072 | 62892 | 2.58E-10 | 0.001 | 39.585 |
| rs7177055 | A | G | 0.0647 | 0.0079 | 62892 | 2.75E-16 | 0.001 | 67.074 |
| rs4502156 | C | T | -0.0411 | 0.0073 | 62892 | 1.66E-08 | 0.001 | 31.698 |
| rs244415 | A | G | -0.0467 | 0.0079 | 62892 | 3.88E-09 | 0.001 | 34.945 |
| rs7185735 | G | A | 0.1056 | 0.0073 | 62892 | 1.59E-47 | 0.003 | 209.258 |
| rs77258096 | A | C | -0.1171 | 0.0134 | 62892 | 1.78E-18 | 0.001 | 76.367 |
| rs2925979 | C | T | -0.0534 | 0.0078 | 62892 | 9.06E-12 | 0.001 | 46.870 |
| rs9940149 | A | G | -0.058 | 0.0095 | 62892 | 9.29E-10 | 0.001 | 37.274 |
| rs17631783 | T | C | -0.0487 | 0.0089 | 62892 | 3.95E-08 | 0.000 | 29.942 |
| rs17405722 | A | G | 0.087 | 0.0146 | 62892 | 2.28E-09 | 0.001 | 35.509 |
| rs302864 | A | G | 0.071 | 0.0127 | 62892 | 2.46E-08 | 0.000 | 31.254 |
| rs9911983 | T | C | 0.0397 | 0.0073 | 62892 | 4.82E-08 | 0.000 | 29.576 |
| rs8068804 | A | G | 0.0587 | 0.0078 | 62892 | 4.41E-14 | 0.001 | 56.635 |
| rs12945601 | C | T | -0.048 | 0.008 | 62892 | 1.72E-09 | 0.001 | 36.000 |
| rs9894220 | G | A | -0.0585 | 0.0079 | 62892 | 1.52E-13 | 0.001 | 54.835 |

| | | | | | | | | | |
|---|---|---|---|---|---|---|---|---|---|
| | rs12970134 | A | G | 0.0555 | 0.008 | 62892 | 5.31E-12 | 0.001 | 48.129 |
| | rs7240767 | C | T | 0.0451 | 0.0081 | 62892 | 2.16E-08 | 0.000 | 31.002 |
| | rs8108269 | G | T | 0.0644 | 0.0079 | 62892 | 3.11E-16 | 0.001 | 66.453 |
| | rs10401969 | C | T | 0.0921 | 0.0133 | 62892 | 4.13E-12 | 0.001 | 47.953 |
| | rs6515236 | C | A | -0.0504 | 0.0091 | 62892 | 3.34E-08 | 0.000 | 30.675 |
| | rs6059662 | G | A | 0.0446 | 0.0079 | 62892 | 1.51E-08 | 0.001 | 31.872 |
| | rs6066138 | A | G | -0.049 | 0.0082 | 62892 | 1.93E-09 | 0.001 | 35.708 |
| | rs4810426 | T | C | 0.0726 | 0.013 | 62892 | 2.15E-08 | 0.000 | 31.188 |
| | rs16988333 | G | A | -0.0745 | 0.013 | 62892 | 9.17E-09 | 0.001 | 32.842 |
| | rs4823182 | G | A | 0.0482 | 0.0077 | 62892 | 3.36E-10 | 0.001 | 39.184 |
| coronary artery disease ($P<5\times10^{-8}$) | rs67180937 | G | T | 0.078807 | 0.0110551 | 42457 | 1.01E-12 | 0.001 | 50.816 |
| | rs6689306 | G | A | -0.056012 | 0.0094061 | 42457 | 2.60E-09 | 0.001 | 35.460 |
| | rs7528419 | G | A | -0.11453 | 0.011482 | 42457 | 1.97E-23 | 0.002 | 99.495 |
| | rs11206510 | C | T | -0.074519 | 0.0133438 | 42457 | 2.34E-08 | 0.001 | 31.187 |
| | rs9970807 | T | C | -0.12575 | 0.016695 | 42457 | 5.00E-14 | 0.001 | 56.734 |
| | rs7568458 | A | T | 0.059618 | 0.0095093 | 42457 | 3.62E-10 | 0.001 | 39.306 |
| | rs115654617 | A | C | 0.137846 | 0.0158314 | 42457 | 3.12E-18 | 0.002 | 75.814 |
| | rs17678683 | G | T | 0.098786 | 0.0166548 | 42457 | 3.00E-09 | 0.001 | 35.181 |
| | rs16986953 | A | G | 0.08516 | 0.0150265 | 42457 | 1.45E-08 | 0.001 | 32.119 |
| | rs515135 | C | T | 0.067499 | 0.0121924 | 42457 | 3.09E-08 | 0.001 | 30.649 |
| | rs1199338 | C | A | 0.073596 | 0.0124987 | 42457 | 3.90E-09 | 0.001 | 34.672 |
| | rs4593108 | G | C | -0.07083 | 0.0115558 | 42457 | 8.82E-10 | 0.001 | 37.569 |
| | rs17087335 | T | G | 0.060764 | 0.0111159 | 42457 | 4.59E-08 | 0.001 | 29.882 |
| | rs72689147 | T | G | -0.068558 | 0.0117905 | 42457 | 6.07E-09 | 0.001 | 33.811 |
| | rs56336142 | C | T | -0.066813 | 0.0118763 | 42457 | 1.85E-08 | 0.001 | 31.649 |
| | rs12202017 | G | A | -0.066813 | 0.0099612 | 42457 | 1.98E-11 | 0.001 | 44.988 |
| | rs55730499 | T | C | 0.316641 | 0.0242403 | 42457 | 5.39E-39 | 0.004 | 170.631 |
| | rs186696265 | T | C | 0.550351 | 0.0481949 | 42457 | 3.35E-30 | 0.003 | 130.400 |
| | rs9349379 | G | A | 0.131836 | 0.0096527 | 42457 | 1.81E-42 | 0.004 | 186.539 |
| | rs3918226 | T | C | 0.133315 | 0.0221275 | 42457 | 1.69E-09 | 0.001 | 36.299 |
| | rs2107595 | A | G | 0.073415 | 0.0112951 | 42457 | 8.05E-11 | 0.001 | 42.246 |
| | rs11556924 | T | C | -0.072569 | 0.0110605 | 42457 | 5.34E-11 | 0.001 | 43.048 |

| | | | | | | | | | |
|---|---|---|---|---|---|---|---|---|---|
| | rs2891168 | G | A | 0.193401 | 0.0091877 | 42457 | 2.29E-98 | 0.010 | 443.102 |
| | rs2487928 | A | G | 0.062633 | 0.0095049 | 42457 | 4.41E-11 | 0.001 | 43.422 |
| | rs1870634 | G | T | 0.075878 | 0.0097113 | 42457 | 5.55E-15 | 0.001 | 61.049 |
| | rs11191416 | G | T | -0.079249 | 0.0135252 | 42457 | 4.65E-09 | 0.001 | 34.332 |
| | rs1412444 | T | C | 0.066812 | 0.0096809 | 42457 | 5.15E-12 | 0.001 | 47.630 |
| | rs2128739 | C | A | -0.065565 | 0.0100568 | 42457 | 7.05E-11 | 0.001 | 42.503 |
| | rs10840293 | A | G | 0.054714 | 0.009619 | 42457 | 1.28E-08 | 0.001 | 32.355 |
| | rs11065979 | T | C | 0.068556 | 0.0107672 | 42457 | 1.93E-10 | 0.001 | 40.540 |
| | rs2681472 | G | A | 0.074114 | 0.0113331 | 42457 | 6.17E-11 | 0.001 | 42.766 |
| | rs4773141 | G | C | 0.069732 | 0.0116482 | 42457 | 2.14E-09 | 0.001 | 35.838 |
| | rs11617955 | A | T | -0.088766 | 0.0161041 | 42457 | 3.55E-08 | 0.001 | 30.382 |
| | rs11838776 | A | G | 0.068566 | 0.0107552 | 42457 | 1.83E-10 | 0.001 | 40.643 |
| | rs10139550 | G | C | 0.05538 | 0.0097569 | 42457 | 1.38E-08 | 0.001 | 32.217 |
| | rs4468572 | C | T | 0.077234 | 0.0095277 | 42457 | 4.44E-16 | 0.002 | 65.711 |
| | rs56062135 | T | C | -0.069743 | 0.0118937 | 42457 | 4.52E-09 | 0.001 | 34.385 |
| | rs8042271 | A | G | -0.096711 | 0.0175662 | 42457 | 3.68E-08 | 0.001 | 30.311 |
| | rs7212798 | C | T | 0.079961 | 0.0142216 | 42457 | 1.88E-08 | 0.001 | 31.613 |
| | rs663129 | A | G | 0.058163 | 0.0105173 | 42457 | 3.20E-08 | 0.001 | 30.583 |
| | rs4420638 | G | A | 0.091906 | 0.0140977 | 42457 | 7.07E-11 | 0.001 | 42.500 |
| | rs41290120 | A | G | -0.181026 | 0.0281987 | 42457 | 1.37E-10 | 0.001 | 41.212 |
| | rs56289821 | A | G | -0.13361 | 0.0170415 | 42457 | 4.44E-15 | 0.001 | 61.470 |
| | rs28451064 | A | G | 0.127571 | 0.015952 | 42457 | 1.33E-15 | 0.002 | 63.955 |
| | rs180803 | T | G | -0.180923 | 0.0283062 | 42457 | 1.64E-10 | 0.001 | 40.853 |
| genus Lachnoclostridium id.11308 ($P<1\times10^{-5}$) | rs12566975 | T | C | -0.0468097 | 0.0105787 | 14306 | 9.57194E-06 | 0.001 | 19.580 |
| | rs1528479 | A | G | 0.0497799 | 0.0111919 | 14306 | 9.63984E-06 | 0.001 | 19.783 |
| | rs615997 | T | C | 0.0511752 | 0.0106491 | 14306 | 2.0268E-06 | 0.002 | 23.094 |
| | rs62285313 | A | G | 0.0864203 | 0.0181565 | 14306 | 1.58332E-06 | 0.002 | 22.655 |
| | rs1031599 | T | G | 0.078627 | 0.0175644 | 14306 | 6.31379E-06 | 0.001 | 20.039 |
| | rs3821998 | C | A | -0.0864066 | 0.0192519 | 14306 | 6.72048E-06 | 0.001 | 20.144 |
| | rs4738679 | A | G | 0.0520267 | 0.011404 | 14306 | 4.41754E-06 | 0.001 | 20.813 |
| | rs1997204 | C | T | 0.108075 | 0.0242022 | 14306 | 5.97077E-06 | 0.001 | 19.941 |
| | rs62028349 | G | C | 0.0469989 | 0.0105971 | 14306 | 9.17044E-06 | 0.001 | 19.670 |

| | | | | | | | | | |
|---|---|---|---|---|---|---|---|---|---|
| | rs72829893 | G | T | 0.117472 | 0.0268103 | 14306 | 5.57763E-06 | 0.001 | 19.198 |
| | rs78068103 | A | G | 0.0886199 | 0.0194248 | 14306 | 3.66522E-06 | 0.001 | 20.814 |
| | rs2385421 | A | G | 0.0746186 | 0.0180734 | 14306 | 7.13724E-06 | 0.001 | 17.046 |
| | rs789029 | C | T | -0.0641288 | 0.0137974 | 14306 | 3.75327E-06 | 0.002 | 21.603 |
| | rs6112314 | A | C | -0.0561715 | 0.0108174 | 14306 | 2.43215E-07 | 0.002 | 26.964 |
| genus Streptococcus id.1853 ($P<1\times10^{-5}$) | rs11720390 | G | A | 0.107024 | 0.0228121 | 14306 | 3.59484E-06 | 0.002 | 22.011 |
| | rs6806351 | T | C | -0.0633829 | 0.0136647 | 14306 | 4.93867E-06 | 0.002 | 21.515 |
| | rs57646748 | G | A | -0.0907696 | 0.0200344 | 14306 | 5.47545E-06 | 0.001 | 20.527 |
| | rs10028567 | C | T | -0.0921167 | 0.0191881 | 14306 | 7.30348E-06 | 0.002 | 23.047 |
| | rs395407 | C | G | 0.0792781 | 0.0173697 | 14306 | 4.36506E-06 | 0.001 | 20.832 |
| | rs77558518 | A | G | -0.103999 | 0.0229714 | 14306 | 4.70858E-06 | 0.001 | 20.497 |
| | rs11764382 | A | G | -0.0695345 | 0.0143671 | 14306 | 1.28632E-06 | 0.002 | 23.424 |
| | rs17708276 | A | G | -0.0793955 | 0.0170628 | 14306 | 3.04096E-06 | 0.002 | 21.652 |
| | rs10448310 | A | G | -0.0517935 | 0.0111324 | 14306 | 3.30704E-06 | 0.002 | 21.646 |
| | rs71481756 | T | G | 0.0931048 | 0.0207949 | 14306 | 6.51478E-06 | 0.001 | 20.046 |
| | rs7916711 | A | G | 0.102891 | 0.0217362 | 14306 | 0.000002717 | 0.002 | 22.407 |
| | rs1918540 | A | G | -0.059639 | 0.0128148 | 14306 | 2.44068E-06 | 0.002 | 21.659 |
| | rs11110281 | T | C | -0.137519 | 0.0227398 | 14306 | 2.58315E-09 | 0.003 | 36.572 |
| | rs2370083 | G | T | -0.0816836 | 0.0185851 | 14306 | 9.75237E-06 | 0.001 | 19.317 |
| | rs72739637 | A | G | 0.0959942 | 0.0193213 | 14306 | 1.03307E-06 | 0.002 | 24.684 |
| | rs6563952 | C | G | -0.0827344 | 0.0180035 | 14306 | 5.8213E-06 | 0.001 | 21.118 |
| | rs4968759 | A | G | -0.0515109 | 0.0112068 | 14306 | 3.7812E-06 | 0.001 | 21.127 |
| | rs9903102 | C | A | -0.0709483 | 0.0155275 | 14306 | 4.17994E-06 | 0.001 | 20.878 |
| genus Actinomyces id.423 ($P<1\times10^{-5}$) | rs71315246 | A | G | -0.0969809 | 0.021925 | 14306 | 9.82969E-06 | 0.001 | 19.566 |
| | rs34583783 | G | T | 0.126596 | 0.0268461 | 14306 | 4.48528E-06 | 0.002 | 22.237 |
| | rs4073240 | G | A | 0.0749687 | 0.0167368 | 14306 | 7.94273E-06 | 0.001 | 20.064 |
| | rs35011108 | A | G | 0.232634 | 0.0512044 | 14306 | 6.33826E-06 | 0.001 | 20.641 |
| | rs4146653 | G | A | 0.0985224 | 0.0214182 | 14306 | 4.49645E-06 | 0.001 | 21.159 |
| | rs10787984 | G | C | 0.094316 | 0.0213513 | 14306 | 9.62299E-06 | 0.001 | 19.513 |
| | rs7915461 | C | T | -0.18776 | 0.0401636 | 14306 | 5.91984E-06 | 0.002 | 21.855 |
| | rs2715439 | T | C | -0.0746684 | 0.0164822 | 14306 | 6.27004E-06 | 0.001 | 20.523 |
| family Streptococcaceae id.1850 ($P<1\times10^{-5}$) | rs77968078 | G | A | -0.0993013 | 0.0224788 | 14306 | 7.93341E-06 | 0.001 | 19.515 |

| | | | | | | | | | |
|---|---|---|---|---|---|---|---|---|---|
| | rs76717940 | T | A | 0.150606 | 0.0334079 | 14306 | 3.08937E-06 | 0.001 | 20.323 |
| | rs6806351 | T | C | -0.0619209 | 0.0135744 | 14306 | 6.93793E-06 | 0.001 | 20.808 |
| | rs10028567 | C | T | -0.0934027 | 0.0190343 | 14306 | 3.72495E-06 | 0.002 | 24.079 |
| | rs57646748 | G | A | -0.088021 | 0.019876 | 14306 | 7.88352E-06 | 0.001 | 19.612 |
| | rs395407 | C | G | 0.0826855 | 0.0172536 | 14306 | 1.32559E-06 | 0.002 | 22.967 |
| | rs77558518 | A | G | -0.104239 | 0.022806 | 14306 | 3.72195E-06 | 0.001 | 20.891 |
| | rs957755 | T | G | -0.0642449 | 0.0142702 | 14306 | 7.41515E-06 | 0.001 | 20.268 |
| | rs2952251 | G | A | 0.0639298 | 0.0126525 | 14306 | 3.72237E-07 | 0.002 | 25.530 |
| | rs28718126 | A | G | 0.109069 | 0.0246868 | 14306 | 9.41044E-06 | 0.001 | 19.520 |
| | rs7916711 | A | G | 0.0959639 | 0.021545 | 14306 | 6.32732E-06 | 0.001 | 19.839 |
| | rs16950051 | A | G | 0.107008 | 0.0236973 | 14306 | 5.33814E-06 | 0.001 | 20.391 |
| | rs11110281 | T | C | -0.130554 | 0.0225943 | 14306 | 1.40136E-08 | 0.002 | 33.387 |
| | rs2370083 | G | T | -0.0842751 | 0.0184509 | 14306 | 4.25667E-06 | 0.001 | 20.862 |
| | rs72739637 | A | G | 0.0927983 | 0.0192021 | 14306 | 1.82163E-06 | 0.002 | 23.355 |
| | rs6563952 | C | G | -0.0801931 | 0.0178576 | 14306 | 8.70583E-06 | 0.001 | 20.166 |
| | rs35344081 | G | A | 0.0609349 | 0.0129703 | 14306 | 2.63846E-06 | 0.002 | 22.072 |
| | rs9903102 | C | A | -0.0693015 | 0.0154096 | 14306 | 4.91802E-06 | 0.001 | 20.226 |
| | rs4968759 | A | G | -0.0544035 | 0.0111271 | 14306 | 8.91887E-07 | 0.002 | 23.905 |
| unknown genus id.2041 ($P<1\times10^{-5}$) | rs1032598 | G | A | -0.0886426 | 0.0189039 | 14306 | 4.07587E-06 | 0.002 | 21.988 |
| | rs16843660 | A | G | 0.234697 | 0.04907 | 14306 | 1.75344E-06 | 0.002 | 22.876 |
| | rs11941716 | A | G | 0.101243 | 0.0224414 | 14306 | 9.0663E-06 | 0.001 | 20.353 |
| | rs249459 | A | G | 0.0737341 | 0.0165018 | 14306 | 8.12307E-06 | 0.001 | 19.965 |
| | rs553072 | G | A | 0.109193 | 0.0230198 | 14306 | 3.69097E-06 | 0.002 | 22.500 |
| | rs1962916 | G | A | -0.0737876 | 0.0162232 | 14306 | 6.13847E-06 | 0.001 | 20.687 |
| | rs35703006 | G | T | 0.0926669 | 0.0190779 | 14306 | 9.00762E-07 | 0.002 | 23.593 |
| | rs921383 | G | A | 0.0723153 | 0.0159706 | 14306 | 7.72894E-06 | 0.001 | 20.503 |
| | rs2651663 | A | G | -0.0762556 | 0.0168635 | 14306 | 5.64144E-06 | 0.001 | 20.448 |
| | rs2336448 | T | C | 0.0773742 | 0.0160883 | 14306 | 1.42899E-06 | 0.002 | 23.130 |
| | rs7187855 | A | C | 0.199941 | 0.0418308 | 14306 | 2.20602E-06 | 0.002 | 22.846 |
| | rs6514318 | T | C | 0.128198 | 0.0281765 | 14306 | 5.37675E-06 | 0.001 | 20.701 |
| genus Oxalobacter id.2978 ($P<1\times10^{-5}$) | rs4428215 | G | A | 0.130293 | 0.0242237 | 14306 | 7.51069E-08 | 0.002 | 28.931 |
| | rs36057338 | G | T | 0.207847 | 0.0421439 | 14306 | 8.79812E-07 | 0.002 | 24.323 |

| | | | | | | | | | |
|---|---|---|---|---|---|---|---|---|---|
| | rs1569853 | T | C | -0.138078 | 0.0296981 | 14306 | 3.64502E-06 | 0.002 | 21.617 |
| | rs6993398 | G | A | 0.127217 | 0.0278855 | 14306 | 7.12771E-06 | 0.001 | 20.813 |
| | rs10464997 | G | A | 0.137691 | 0.0294804 | 14306 | 3.29754E-06 | 0.002 | 21.814 |
| | rs12002250 | A | C | 0.217122 | 0.0466317 | 14306 | 1.41504E-06 | 0.002 | 21.679 |
| | rs736744 | T | C | -0.117882 | 0.0211262 | 14306 | 2.57472E-08 | 0.002 | 31.135 |
| | rs3862635 | C | T | -0.172142 | 0.0394026 | 14306 | 9.18692E-06 | 0.001 | 19.086 |
| | rs11108500 | A | G | -0.199099 | 0.0427327 | 14306 | 3.74283E-06 | 0.002 | 21.708 |
| | rs111966731 | T | C | 0.213114 | 0.047162 | 14306 | 7.29861E-06 | 0.001 | 20.419 |
| | rs6071435 | T | A | -0.105512 | 0.021489 | 14306 | 1.07431E-06 | 0.002 | 24.109 |
| | rs6000536 | C | T | -0.130992 | 0.0253804 | 14306 | 2.06054E-07 | 0.002 | 26.637 |
| genus Turicibacter id.2162 ($P<1\times10^{-5}$) | rs149744580 | A | G | 0.169883 | 0.0315478 | 14306 | 7.00971E-08 | 0.002 | 28.998 |
| | rs4869133 | G | A | 0.131186 | 0.027197 | 14306 | 2.5537E-06 | 0.002 | 23.267 |
| | rs2221441 | G | C | 0.0710364 | 0.015343 | 14306 | 3.45669E-06 | 0.001 | 21.436 |
| | rs3734633 | G | A | -0.120957 | 0.02683 | 14306 | 5.31912E-06 | 0.001 | 20.325 |
| | rs55756211 | T | C | -0.115115 | 0.0240708 | 14306 | 2.8053E-06 | 0.002 | 22.871 |
| | rs2952020 | A | G | 0.0759019 | 0.0165764 | 14306 | 5.63313E-06 | 0.001 | 20.966 |
| | rs61265175 | G | C | -0.0858591 | 0.0185778 | 14306 | 4.13676E-06 | 0.001 | 21.359 |
| | rs11054680 | T | C | -0.104751 | 0.0226997 | 14306 | 2.30978E-06 | 0.001 | 21.295 |
| | rs4247078 | G | C | -0.0710377 | 0.0155221 | 14306 | 5.46072E-06 | 0.001 | 20.945 |
| | rs11649454 | G | C | 0.0950891 | 0.0203433 | 14306 | 3.26625E-06 | 0.002 | 21.848 |
| | rs7199484 | G | A | -0.0731428 | 0.0160172 | 14306 | 5.7666E-06 | 0.001 | 20.853 |
| | rs12603364 | T | C | 0.110861 | 0.0225598 | 14306 | 8.66603E-07 | 0.002 | 24.148 |
| | rs11666533 | C | T | -0.111689 | 0.0248436 | 14306 | 7.37106E-06 | 0.001 | 20.211 |
| | rs2834977 | T | C | -0.0959995 | 0.0208261 | 14306 | 3.95585E-06 | 0.001 | 21.248 |
| genus Butyricicoccus id.2055 ($P<1\times10^{-5}$) | rs12034718 | G | A | -0.0701199 | 0.0158213 | 14306 | 9.57679E-06 | 0.001 | 19.643 |
| | rs10084203 | G | A | -0.0549699 | 0.0123563 | 14306 | 8.58638E-06 | 0.001 | 19.791 |
| | rs56221232 | T | C | 0.0828027 | 0.0167401 | 14306 | 7.61939E-07 | 0.002 | 24.467 |
| | rs2017189 | T | G | 0.0506956 | 0.011024 | 14306 | 3.87258E-06 | 0.001 | 21.148 |
| | rs62478070 | T | G | 0.224039 | 0.0494959 | 14306 | 5.93772E-06 | 0.001 | 20.488 |
| | rs4962426 | T | G | -0.0614216 | 0.0135979 | 14306 | 7.38482E-06 | 0.001 | 20.403 |
| | rs7322368 | C | T | -0.0815733 | 0.0183167 | 14306 | 5.51785E-06 | 0.001 | 19.834 |
| | rs12585793 | T | C | -0.262206 | 0.0564729 | 14306 | 5.79189E-06 | 0.002 | 21.558 |

| | | | | | | | | | |
|---|---|---|---|---|---|---|---|---|---|
| | rs75238760 | T | A | 0.0619423 | 0.0139942 | 14306 | 6.79704E-06 | 0.001 | 19.592 |
| unknown genus id.2071 ($P<1\times10^{-5}$) | rs4644504 | T | C | -0.0969321 | 0.0216146 | 14306 | 5.81969E-06 | 0.001 | 20.111 |
| | rs11809762 | G | A | -0.0934634 | 0.0190198 | 14306 | 1.68287E-06 | 0.002 | 24.147 |
| | rs11904514 | A | G | 0.109498 | 0.0249839 | 14306 | 7.89951E-06 | 0.001 | 19.208 |
| | rs1809136 | C | G | -0.0994594 | 0.0228638 | 14306 | 8.36989E-06 | 0.001 | 18.923 |
| | rs16823675 | C | T | -0.0767515 | 0.0149973 | 14306 | 2.33346E-07 | 0.002 | 26.191 |
| | rs11684166 | A | G | -0.0769635 | 0.0168349 | 14306 | 3.49116E-06 | 0.001 | 20.900 |
| | rs10200320 | T | C | -0.0641139 | 0.0142769 | 14306 | 5.6607E-06 | 0.001 | 20.167 |
| | rs2898979 | G | C | 0.0901515 | 0.0202199 | 14306 | 7.67291E-06 | 0.001 | 19.879 |
| | rs35740166 | C | T | -0.112246 | 0.0226824 | 14306 | 8.39982E-07 | 0.002 | 24.489 |
| | rs17086536 | C | A | -0.100851 | 0.022432 | 14306 | 3.3638E-06 | 0.001 | 20.213 |
| | rs34985298 | G | A | -0.0623526 | 0.013772 | 14306 | 8.33758E-06 | 0.001 | 20.498 |
| | rs1455639 | A | G | -0.0760307 | 0.0169831 | 14306 | 7.83899E-06 | 0.001 | 20.042 |
| | rs11195523 | C | A | -0.0689278 | 0.0145351 | 14306 | 2.40121E-06 | 0.002 | 22.488 |
| | rs2939766 | A | G | -0.0591611 | 0.013042 | 14306 | 7.01148E-06 | 0.001 | 20.577 |
| | rs76532867 | T | C | 0.112353 | 0.0242364 | 14306 | 2.55859E-06 | 0.001 | 21.490 |
| | rs56975773 | T | A | 0.113282 | 0.0248859 | 14306 | 7.65491E-06 | 0.001 | 20.721 |
| | rs12147596 | C | T | -0.0719818 | 0.0141609 | 14306 | 2.86207E-07 | 0.002 | 25.838 |
| | rs72700702 | T | C | -0.091726 | 0.0189005 | 14306 | 1.59272E-06 | 0.002 | 23.553 |
| | rs72707147 | C | T | 0.110109 | 0.0244644 | 14306 | 6.84022E-06 | 0.001 | 20.257 |
| | rs6007642 | C | T | -0.0791412 | 0.0177958 | 14306 | 9.95543E-06 | 0.001 | 19.777 |
| family Clostridiales vadin BB60 group id.11286 | rs7538034 | T | G | -0.078598 | 0.0165982 | 14306 | 2.36706E-06 | 0.002 | 22.423 |
| ($P<1\times10^{-5}$) | rs6588624 | A | G | 0.0662317 | 0.0138147 | 14306 | 1.79287E-06 | 0.002 | 22.985 |
| | rs13409132 | A | G | -0.165419 | 0.0352154 | 14306 | 4.3723E-06 | 0.002 | 22.065 |
| | rs2191834 | T | G | -0.0746375 | 0.0159136 | 14306 | 2.50196E-06 | 0.002 | 21.998 |
| | rs6755871 | C | G | -0.0613825 | 0.0138976 | 14306 | 9.33061E-06 | 0.001 | 19.508 |
| | rs989682 | A | G | 0.070194 | 0.0155364 | 14306 | 6.84715E-06 | 0.001 | 20.413 |
| | rs10517600 | G | T | -0.0626993 | 0.0139364 | 14306 | 6.82763E-06 | 0.001 | 20.241 |
| | rs34088226 | A | G | -0.117807 | 0.026924 | 14306 | 7.66214E-06 | 0.001 | 19.145 |
| | rs7725895 | A | G | -0.116224 | 0.0240367 | 14306 | 3.94357E-06 | 0.002 | 23.380 |
| | rs66714985 | A | C | 0.116908 | 0.0252447 | 14306 | 4.85333E-06 | 0.001 | 21.446 |
| | rs118104867 | C | T | 0.214464 | 0.0455098 | 14306 | 3.43598E-06 | 0.002 | 22.207 |

| | | | | | | | | | |
|---|---|---|---|---|---|---|---|---|---|
| | rs10904722 | C | T | -0.0672314 | 0.0147123 | 14306 | 5.04836E-06 | 0.001 | 20.883 |
| | rs17121075 | G | A | 0.0769254 | 0.0172234 | 14306 | 7.91425E-06 | 0.001 | 19.948 |
| | rs55682560 | C | T | -0.131519 | 0.0261319 | 14306 | 4.97038E-07 | 0.002 | 25.330 |
| | rs28691777 | C | T | 0.137134 | 0.0266996 | 14306 | 6.95697E-07 | 0.002 | 26.380 |
| | rs7226487 | A | G | -0.0643682 | 0.0138701 | 14306 | 3.58286E-06 | 0.002 | 21.537 |
| | rs9979874 | G | C | -0.0738925 | 0.0150911 | 14306 | 1.05271E-06 | 0.002 | 23.975 |
| unknown genus id.1000000073 ($P<1\times10^{-5}$) | rs6588624 | A | G | 0.0662317 | 0.0138147 | 14306 | 1.79287E-06 | 0.002 | 22.985 |
| | rs7538034 | T | G | -0.078598 | 0.0165982 | 14306 | 2.36706E-06 | 0.002 | 22.423 |
| | rs2191834 | T | G | -0.0746375 | 0.0159136 | 14306 | 2.50196E-06 | 0.002 | 21.998 |
| | rs13409132 | A | G | -0.165419 | 0.0352154 | 14306 | 4.3723E-06 | 0.002 | 22.065 |
| | rs6755871 | C | G | -0.0613825 | 0.0138976 | 14306 | 9.33061E-06 | 0.001 | 19.508 |
| | rs989682 | A | G | 0.070194 | 0.0155364 | 14306 | 6.84715E-06 | 0.001 | 20.413 |
| | rs10517600 | G | T | -0.0626993 | 0.0139364 | 14306 | 6.82763E-06 | 0.001 | 20.241 |
| | rs7725895 | A | G | -0.116224 | 0.0240367 | 14306 | 3.94357E-06 | 0.002 | 23.380 |
| | rs34088226 | A | G | -0.117807 | 0.026924 | 14306 | 7.66214E-06 | 0.001 | 19.145 |
| | rs66714985 | A | C | 0.116908 | 0.0252447 | 14306 | 4.85333E-06 | 0.001 | 21.446 |
| | rs118104867 | C | T | 0.214464 | 0.0455098 | 14306 | 3.43598E-06 | 0.002 | 22.207 |
| | rs10904722 | C | T | -0.0672314 | 0.0147123 | 14306 | 5.04836E-06 | 0.001 | 20.883 |
| | rs17121075 | G | A | 0.0769254 | 0.0172234 | 14306 | 7.91425E-06 | 0.001 | 19.948 |
| | rs55682560 | C | T | -0.131519 | 0.0261319 | 14306 | 4.97038E-07 | 0.002 | 25.330 |
| | rs28691777 | C | T | 0.137134 | 0.0266996 | 14306 | 6.95697E-07 | 0.002 | 26.380 |
| | rs7226487 | A | G | -0.0643682 | 0.0138701 | 14306 | 3.58286E-06 | 0.002 | 21.537 |
| | rs9979874 | G | C | -0.0738925 | 0.0150911 | 14306 | 1.05271E-06 | 0.002 | 23.975 |
| genus Clostridium innocuum group id.14397 ($P<1\times10^{-5}$) | rs6577484 | G | A | 0.160425 | 0.0360857 | 14306 | 8.40601E-06 | 0.001 | 19.764 |
| | rs1948423 | T | A | -0.108859 | 0.023425 | 14306 | 3.49406E-06 | 0.002 | 21.596 |
| | rs40656 | C | T | 0.142664 | 0.0311021 | 14306 | 8.61529E-06 | 0.001 | 21.040 |
| | rs6890185 | C | T | -0.113424 | 0.0233137 | 14306 | 1.12243E-06 | 0.002 | 23.669 |
| | rs4869133 | G | A | -0.180591 | 0.0409505 | 14306 | 7.24453E-06 | 0.001 | 19.448 |
| | rs10074000 | T | C | -0.102648 | 0.0227508 | 14306 | 6.99939E-06 | 0.001 | 20.357 |
| | rs71564433 | T | A | -0.126746 | 0.0274657 | 14306 | 7.8001E-06 | 0.001 | 21.295 |
| | rs10506058 | A | G | 0.0997048 | 0.0221926 | 14306 | 8.92442E-06 | 0.001 | 20.184 |
| | rs77845139 | A | G | -0.114993 | 0.0257186 | 14306 | 8.40621E-06 | 0.001 | 19.992 |

| | | | | | | | | | |
|---|---|---|---|---|---|---|---|---|---|
| | rs61267978 | T | C | 0.14708 | 0.0320875 | 14306 | 5.58509E-06 | 0.001 | 21.010 |
| | rs1942371 | G | A | -0.157938 | 0.034187 | 14306 | 4.0634E-06 | 0.001 | 21.343 |
| class Lentisphaeria id.2250 ($P<1\times10^{-5}$) | rs72640280 | A | G | 0.220207 | 0.0486196 | 14306 | 5.18036E-06 | 0.001 | 20.513 |
| | rs73113483 | T | A | -0.131217 | 0.0288713 | 14306 | 8.66343E-06 | 0.001 | 20.656 |
| | rs2731834 | G | C | -0.109438 | 0.023693 | 14306 | 4.24356E-06 | 0.001 | 21.335 |
| | rs11770843 | C | T | 0.109431 | 0.0234879 | 14306 | 1.9073E-06 | 0.002 | 21.707 |
| | rs62570196 | C | T | -0.21635 | 0.0439866 | 14306 | 1.07924E-06 | 0.002 | 24.192 |
| | rs2031282 | A | G | 0.122368 | 0.0270329 | 14306 | 4.38258E-06 | 0.001 | 20.490 |
| | rs17114848 | G | A | 0.152377 | 0.0324332 | 14306 | 4.05864E-06 | 0.002 | 22.073 |
| | rs1002941 | A | G | -0.105025 | 0.0233484 | 14306 | 8.14836E-06 | 0.001 | 20.234 |
| | rs77599476 | A | G | 0.230292 | 0.0480168 | 14306 | 1.86132E-06 | 0.002 | 23.002 |
| | rs2825714 | A | G | -0.13741 | 0.0289246 | 14306 | 1.72211E-06 | 0.002 | 22.568 |
| order Victivallales id.2254 ($P<1\times10^{-5}$) | rs72640280 | A | G | 0.220207 | 0.0486196 | 14306 | 5.18036E-06 | 0.001 | 20.513 |
| | rs73113483 | T | A | -0.131217 | 0.0288713 | 14306 | 8.66343E-06 | 0.001 | 20.656 |
| | rs2731834 | G | C | -0.109438 | 0.023693 | 14306 | 4.24356E-06 | 0.001 | 21.335 |
| | rs11770843 | C | T | 0.109431 | 0.0234879 | 14306 | 1.9073E-06 | 0.002 | 21.707 |
| | rs62570196 | C | T | -0.21635 | 0.0439866 | 14306 | 1.07924E-06 | 0.002 | 24.192 |
| | rs2031282 | A | G | 0.122368 | 0.0270329 | 14306 | 4.38258E-06 | 0.001 | 20.490 |
| | rs1002941 | A | G | -0.105025 | 0.0233484 | 14306 | 8.14836E-06 | 0.001 | 20.234 |
| | rs17114848 | G | A | 0.152377 | 0.0324332 | 14306 | 4.05864E-06 | 0.002 | 22.073 |
| | rs77599476 | A | G | 0.230292 | 0.0480168 | 14306 | 1.86132E-06 | 0.002 | 23.002 |
| | rs2825714 | A | G | -0.13741 | 0.0289246 | 14306 | 1.72211E-06 | 0.002 | 22.568 |
| genus Bifidobacterium id.436 ($P<1\times10^{-5}$) | rs12022129 | A | G | -0.0619356 | 0.0138937 | 14306 | 7.9965E-06 | 0.001 | 19.872 |
| | rs1961273 | C | T | 0.0674036 | 0.0132319 | 14306 | 3.50865E-07 | 0.002 | 25.949 |
| | rs13020688 | G | A | 0.0562696 | 0.0122617 | 14306 | 4.07258E-06 | 0.001 | 21.059 |
| | rs182549 | T | C | -0.119703 | 0.0127294 | 14306 | 1.2782E-20 | 0.006 | 88.429 |
| | rs62181700 | G | A | -0.0624643 | 0.0131205 | 14306 | 2.17245E-06 | 0.002 | 22.665 |
| | rs4567981 | T | A | 0.0562084 | 0.0117923 | 14306 | 1.92832E-06 | 0.002 | 22.720 |
| | rs55888705 | A | G | 0.0546319 | 0.0121139 | 14306 | 6.67022E-06 | 0.001 | 20.339 |
| | rs4957061 | T | C | 0.0534239 | 0.0117431 | 14306 | 5.77936E-06 | 0.001 | 20.697 |
| | rs73797465 | T | G | -0.0953566 | 0.0209236 | 14306 | 4.38157E-06 | 0.001 | 20.770 |
| | rs76671854 | C | G | -0.0846055 | 0.0184003 | 14306 | 3.95667E-06 | 0.001 | 21.142 |

| | | | | | | | | |
|---|---|---|---|---|---|---|---|---|
| rs857444 | C | T | 0.0558234 | 0.0121219 | 14306 | 0.000003571 | 0.001 | 21.208 |
| rs2686790 | C | T | -0.070741 | 0.0157926 | 14306 | 7.49894E-06 | 0.001 | 20.065 |
| rs2491158 | A | G | -0.0712624 | 0.015983 | 14306 | 8.04711E-06 | 0.001 | 19.879 |
| rs10841473 | G | C | -0.0624207 | 0.0129438 | 14306 | 1.6452E-06 | 0.002 | 23.256 |
| rs7322849 | T | C | 0.112428 | 0.0201813 | 14306 | 1.08368E-08 | 0.002 | 31.035 |
| rs540489 | T | G | -0.0637641 | 0.0138746 | 14306 | 5.19457E-06 | 0.001 | 21.121 |
| rs75344046 | C | T | 0.232354 | 0.0505979 | 14306 | 4.86351E-06 | 0.001 | 21.088 |
| rs5746486 | T | C | -0.0536216 | 0.0120801 | 14306 | 8.99953E-06 | 0.001 | 19.703 |

SNPs, single nucleotide polymorphisms; EA, effect allele; OA, other allele; Beta, effect estimate; SE, standard error; $R^2 = (beta^2) / (se^2 \times N + beta^2)$; $F = beta^2 / se^2$.

**Table S6. MR Power calculation for detecting significant (P < 0.05) causal effect of gut microbiota on the risk of T2D/CAD, and T2D/CAD on the risk of gut microbiota.**

| Expoure | Outcome | NSNPs | $R^2$ | Power | |
|---|---|---|---|---|---|
| | | | | beta=0.1 | beta=0.2 |
| T2D | genus Butyrivibrio id.1993 | 124 | 0.143 | 1.00 | 1.00 |
| | genus Catenibacterium id.2153 | 114 | 0.134 | 1.00 | 1.00 |
| | genus Olsenella id.822 | 124 | 0.143 | 1.00 | 1.00 |
| | family Oxalobacteraceae id.2966 | 125 | 0.143 | 1.00 | 1.00 |
| CAD | genus Erysipelotrichaceae UCG003 id.11384 | 14 | 0.013 | 0.82 | 1.00 |
| | genus Veillonella id.2198 | 36 | 0.053 | 1.00 | 1.00 |
| | genus Butyricicoccus id.2055 | 36 | 0.053 | 1.00 | 1.00 |
| | family Christensenellaceae id.1866 | 10 | 0.011 | 0.58 | 0.99 |
| | genus Ruminococcaceae UCG004 id.11362 | 36 | 0.053 | 1.00 | 1.00 |
| | genus Haemophilus id.3698 | 36 | 0.053 | 1.00 | 1.00 |
| | class Gammaproteobacteria id.3303 | 36 | 0.053 | 1.00 | 1.00 |
| | family Prevotellaceae id.960 | 36 | 0.053 | 1.00 | 1.00 |
| | genus Coprococcus1 id.11301 | 36 | 0.053 | 1.00 | 1.00 |
| | genus Lachnospiraceae UCG008 id.11328 | 35 | 0.052 | 1.00 | 1.00 |
| | genus Family XIII UCG001 id.11294 | 36 | 0.053 | 1.00 | 1.00 |
| | genus Methanobrevibacter id.123 | 34 | 0.048 | 1.00 | 1.00 |
| | genus Lachnospiraceae UCG010 id.11330 | 36 | 0.053 | 1.00 | 1.00 |
| | class Methanobacteria id.119 | 34 | 0.048 | 1.00 | 1.00 |
| | family Methanobacteriaceae id.121 | 34 | 0.048 | 1.00 | 1.00 |
| | order Methanobacteriales id.120 | 34 | 0.048 | 1.00 | 1.00 |
| | family Lachnospiraceae id.1987 | 36 | 0.053 | 1.00 | 1.00 |
| | family Pasteurellaceae id.3689 | 36 | 0.053 | 1.00 | 1.00 |
| | order Pasteurellales id.3688 | 36 | 0.053 | 1.00 | 1.00 |
| | genus Prevotella9 id.11183 | 36 | 0.053 | 1.00 | 1.00 |

| Outcome | Expoure | NSNPs | $R^2$ | Power | |
|---|---|---|---|---|---|
| | | | | beta=0.1 | beta=0.2 |
| CAD | genus Oxalobacter id.2978 | 11 | 0.018 | 0.36 | 0.91 |
| | genus Turicibacter id.2162 | 10 | 0.016 | 0.33 | 0.87 |
| | genus Butyricicoccus id.2055 | 8 | 0.120 | 0.26 | 0.76 |
| | unknown genus id.2071 | 16 | 0.240 | 0.46 | 0.97 |
| | family Clostridiales vadin BB60 group id.11286 | 15 | 0.260 | 0.49 | 0.98 |
| | unknown genus id.1000000073 | 15 | 0.230 | 0.45 | 0.96 |
| | genus Clostridium innocuum group id.14397 | 9 | 0.130 | 0.28 | 0.79 |
| | class Lentisphaeria id.2250 | 8 | 0.120 | 0.26 | 0.76 |
| | order Victivallales id.2254 | 8 | 0.120 | 0.26 | 0.76 |
| | genus Bifidobacterium id.436 | 14 | 0.260 | 0.49 | 0.98 |
| T2D | genus Lachnoclostridium id.11308 | 8 | 0.012 | 0.26 | 0.76 |
| | genus Streptococcus id.1853 | 11 | 0.018 | 0.36 | 0.91 |
| | genus Actinomyces id.423 | 5 | 0.007 | 0.17 | 0.53 |
| | family Streptococcaceae id.1850 | 13 | 0.020 | 0.40 | 0.93 |
| | unknown genus id.2041 | 6 | 0.009 | 0.21 | 0.64 |

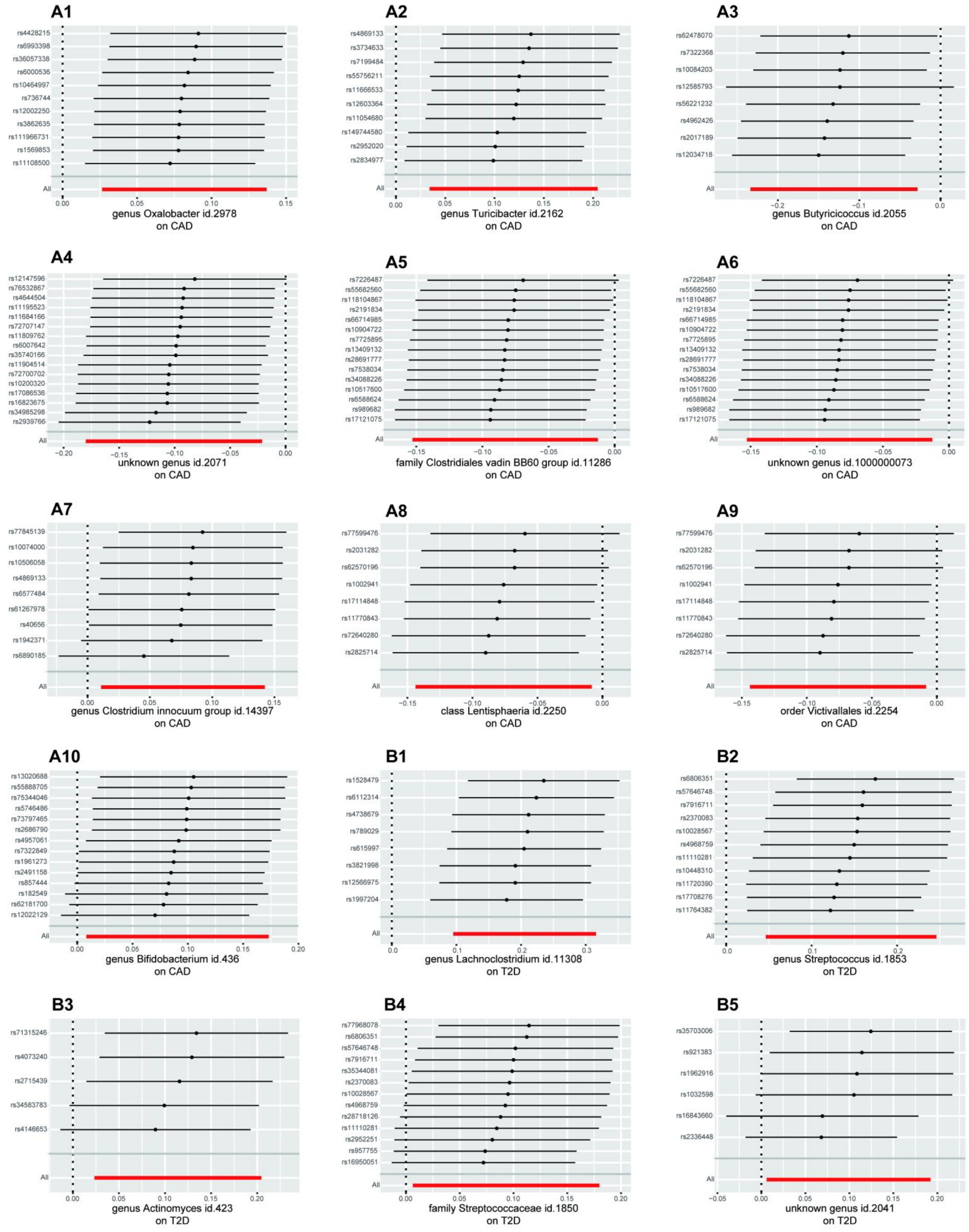


**Figure S1. Leave-one-out analysis of inverse-variance weighted (IVW) estimates between genetically determined gut microbiota traits ($p < 1 \times 10^{-5}$) and Type 2 diabetes (T2D), as well as coronary artery disease (CAD).**

Black dots indicate the IVW estimates (raw beta) after leaving a SNP in the calculation. The red dots indicate the pooled IVW estimates (raw beta). The horizontal line indicates the range of 95% confidence intervals.

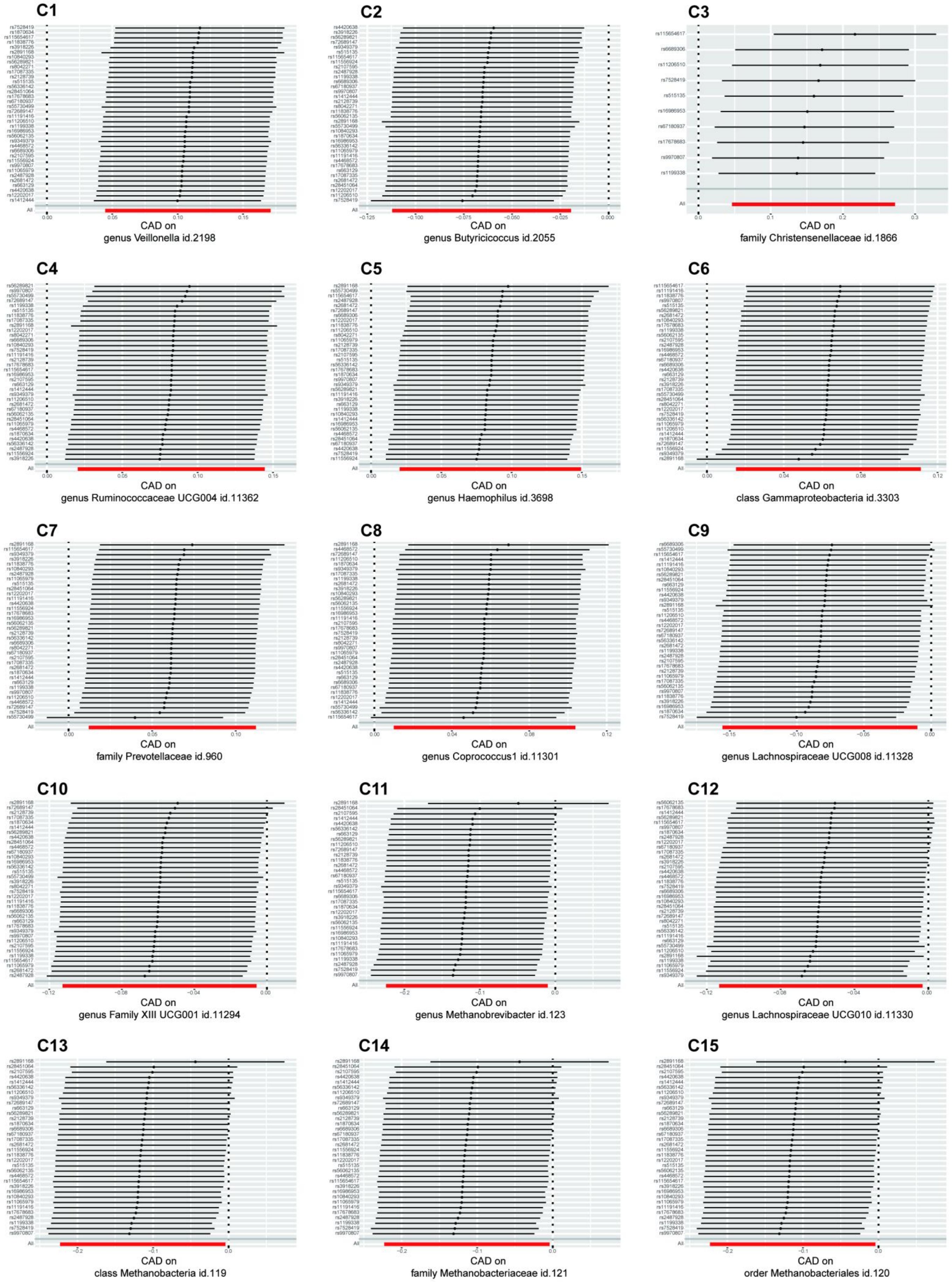
C1
CAD on
genus Veillonella id.2198
C2
CAD on
genus Butyricicoccus id.2055
C3
CAD on
family Christensenellaceae id.1866
C4
CAD on
genus Ruminococcaceae UCG004 id.11362
C5
CAD on
genus Haemophilus id.3698
C6
CAD on
class Gammaproteobacteria id.3303
C7
CAD on
family Prevotellaceae id.960
C8
CAD on
genus Coprococcus1 id.11301
C9
CAD on
genus Lachnospiraceae UCG008 id.11328
C10
CAD on
genus Family XIII UCG001 id.11294
C11
CAD on
genus Methanobrevibacter id.123
C12
CAD on
genus Lachnospiraceae UCG010 id.11330
C13
CAD on
class Methanobacteria id.119
C14
CAD on
family Methanobacteriaceae id.121
C15
CAD on
order Methanobacteriales id.120

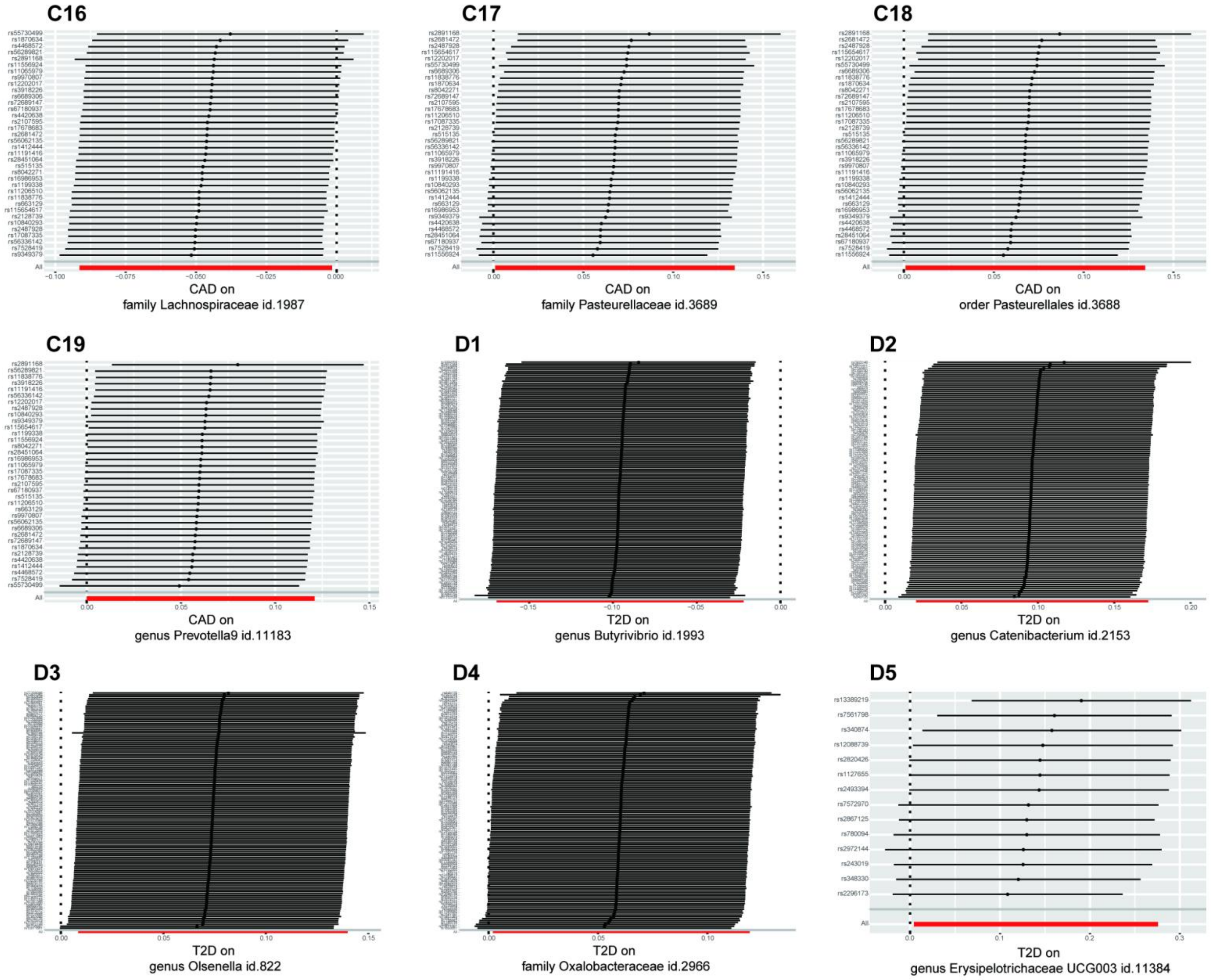


**Figure S2. Leave-one-out analysis of inverse-variance weighted (IVW) estimates between genetically determined type 2 diabetes (T2D) traits ($P < 5 \times 10^{-8}$), as well as coronary artery disease (CAD) traits ($P < 5 \times 10^{-8}$), and gut microbiota.**

Black dots indicate the IVW estimates (raw beta) after leaving a SNP in the calculation. The red dots indicate the pooled IVW estimates (raw beta). The horizontal line indicates the range of 95% confidence intervals.

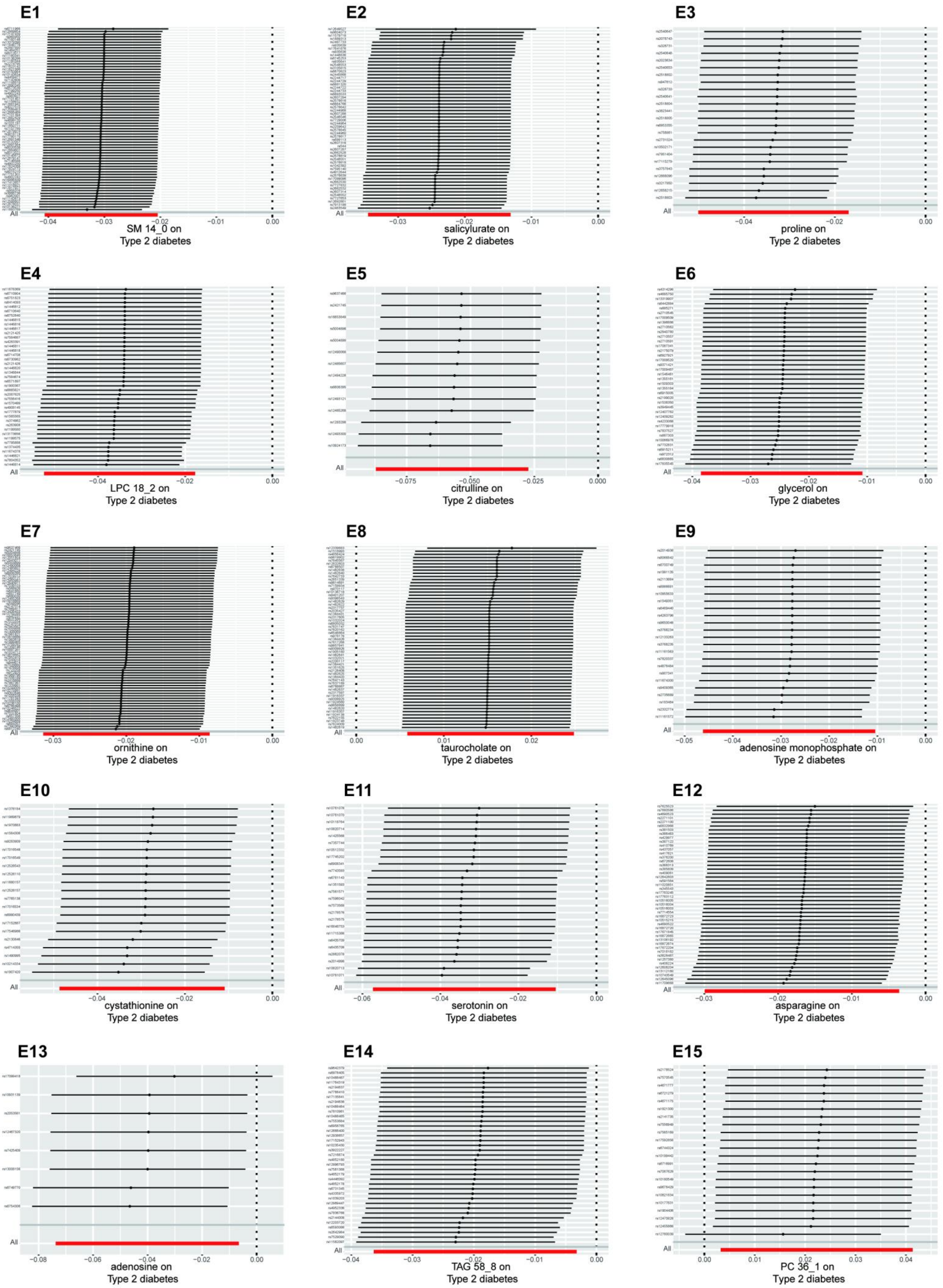

E1
All
-0.04 -0.03 -0.02 -0.01 0.00
SM 14_0 on
Type 2 diabetes
E2
All
-0.03 -0.02 -0.01 0.00
salicylurate on
Type 2 diabetes
E3
All
-0.04 -0.02 0.00
proline on
Type 2 diabetes
E4
All
-0.04 -0.02 0.00
LPC 18_2 on
Type 2 diabetes
E5
All
-0.075 -0.050 -0.025 0.000
citrulline on
Type 2 diabetes
E6
All
-0.04 -0.03 -0.02 -0.01 0.00
glycerol on
Type 2 diabetes
E7
All
-0.03 -0.02 -0.01 0.00
ornithine on
Type 2 diabetes
E8
All
0.00 0.01 0.02
taurocholate on
Type 2 diabetes
E9
All
-0.05 -0.04 -0.03 -0.02 -0.01 0.00
adenosine monophosphate on
Type 2 diabetes
E10
All
-0.04 -0.02 0.00
cystathionine on
Type 2 diabetes
E11
All
-0.06 -0.04 -0.02 0.00
serotonin on
Type 2 diabetes
E12
All
-0.03 -0.02 -0.01 0.00
asparagine on
Type 2 diabetes
E13
All
-0.08 -0.06 -0.04 -0.02 0.00
adenosine on
Type 2 diabetes
E14
All
-0.04 -0.03 -0.02 -0.01 0.00
TAG 58_8 on
Type 2 diabetes
E15
All
0.00 0.01 0.02 0.03 0.04
PC 36_1 on
Type 2 diabetes

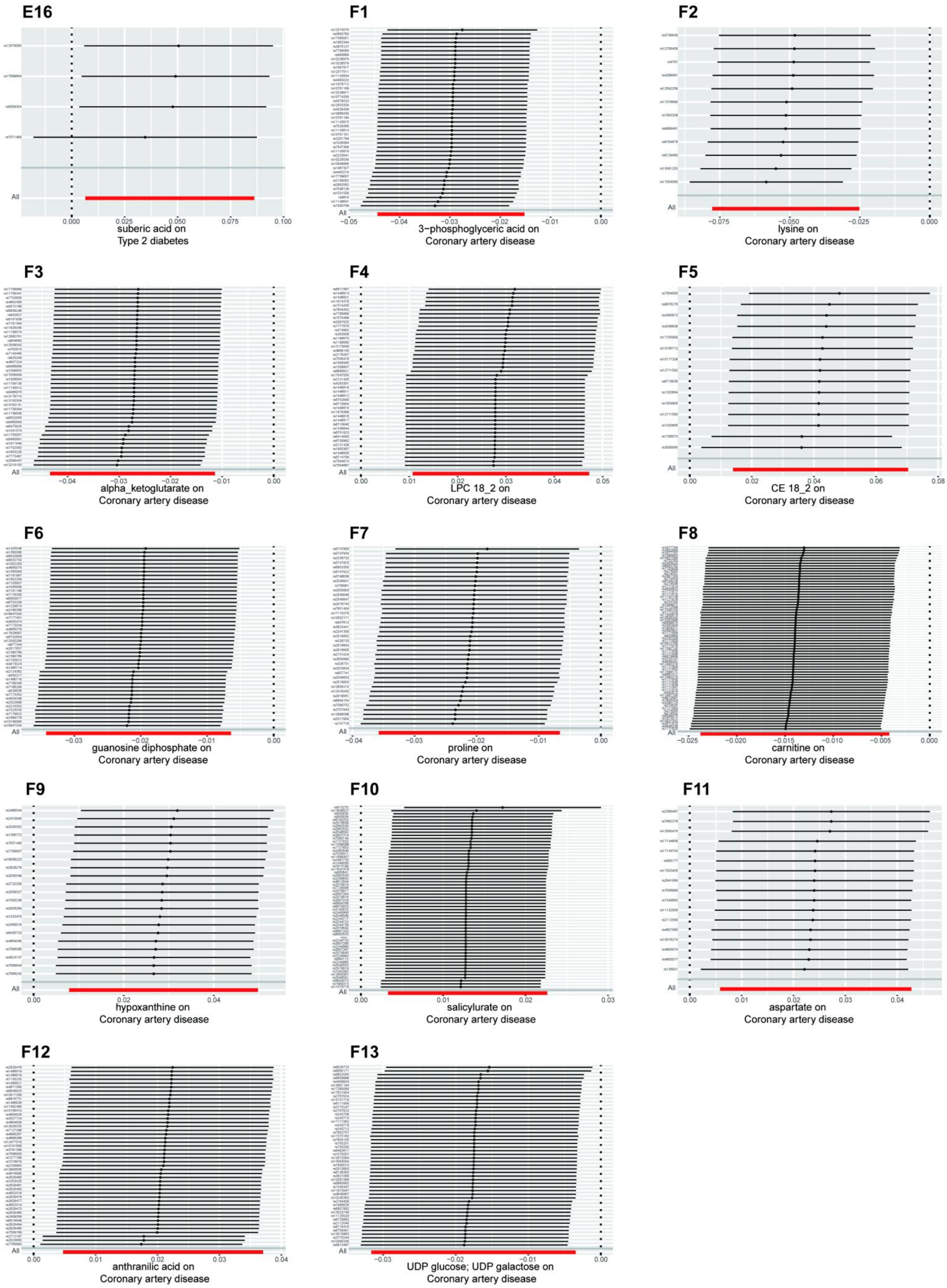


**Figure S3.** Leave-one-out analysis of inverse-variance weighted (IVW) estimates between genetically determined metabolites traits ($p < 1 \times 10^{-5}$) and Type 2 diabetes (T2D), as well as coronary artery disease (CAD).

Black dots indicate the IVW estimates (raw beta) after leaving a SNP in the calculation. The red dots indicate the pooled IVW estimates (raw beta). The horizontal line indicates the range of 95% confidence intervals.
